# Comprehensive Profiling of Free Proteinogenic and Non-Proteinogenic Amino Acids in Common Legumes Using LC-QToF: Targeted and Non-Targeted Approaches

**DOI:** 10.3390/foods14040611

**Published:** 2025-02-12

**Authors:** Bharathi Avula, Kumar Katragunta, Iffat Parveen, Kiran Kumar Tatapudi, Amar G. Chittiboyina, Yan-Hong Wang, Ikhlas A. Khan

**Affiliations:** 1 National Center for Natural Products Research, School of Pharmacy, University of Mississippi, University, MS 38677, USA; kkatragu@olemiss.edu (K.K.); iparveen@olemiss.edu (I.P.); kktatapu@olemiss.edu (K.K.T.); amar@olemiss.edu (A.G.C.); wangyh@olemiss.edu (Y.-H.W.); 2 Division of Pharmacognosy, Department of BioMolecular Sciences, School of Pharmacy, University of Mississippi, University, MS 38677, USA

**Keywords:** plant-based proteins, non-proteinogenic amino acids, derivatization, LC-QToF-MS, soluble protein profiles

## Abstract

Legumes, a dietary staple for centuries, have seen an influx of conventional and unconventional varieties to cater to human care conscious consumers. These legumes often undergo pretreatments like baking, soaking, or boiling to mitigate the presence of non-proteinogenic amino acids (NPAAs) and reduce associated health risks. The recent tara flour health scare, linked to the NPAA baikiain, emphasizes the need for robust analytical methods to ensure the safety and quality of both traditional and novel plant-based protein alternatives. While traditional techniques provide insights into protein and non-proteinogenic amino acid profiles, modern liquid chromatography-mass spectrometry (LC-MS) offers superior sensitivity and specificity for NPAA detection. This study employed an LC-QToF method with MS/MS analysis to comprehensively map the distribution of free NPAAs and proteinogenic amino acids (PAAs) in various legume samples. A total of 47 NPAAs and 20 PAAs were identified across the legume samples, with at least 7–14 NPAAs detected in each sample. Sulfur-containing NPAAs, such as *S*-methyl-*L*-cysteine, γ-glutamyl-*S*-methyl cysteine, and *S*-methyl homoglutathione, were predominantly found in *Phaseolus* and *Vigna* species. Cysteine and methionine were the sulfur-containing PAAs identified. Gel electrophoresis and soluble protein quantification were also conducted to understand legume protein composition holistically. This orthogonal approach provides a valuable tool for ensuring the overall quality of plant-based proteins and may aid in investigating food poisoning or outbreaks related to such products.

## 1. Introduction

*Fabaceae*, the legume family, is one of the largest plant families, with six sub-families cultivated globally for millennia as a staple food and significant livestock feed. Common examples include *Phaseolus vulgaris* L. (common bean), *Lens culinaris* L. (lentils), *Cicer arietinum* (chickpea), *Cajanus cajan* L. (pigeon pea), *Vigna unguiculata* (cow pea), *Pisum sativum* L. (green pea), as well as *Glycine max* L. (soy), *Arachis hypogaea* L. (peanut), and *Medicago sativa* (alfalfa). With their nitrogen-fixing ability, legumes are rich in alkaloids, amines, cyanogenic glucosides, and proteins consisting of common proteinogenic amino acids (PAAs) and non-proteinogenic amino acids (NPAAs). Other secondary metabolites such as phenolics, polyketides, and terpenoids are also part of legume chemical composition [1].

Legumes, a major source of non-animal protein worldwide, typically contain all 20 proteinogenic amino acids [2]. However, they also include NPAAs, which differ from PAAs in their inability to be incorporated into proteins. Certain NPAAs specific to particular legume species can serve as valuable chemical markers of product authenticity and the presence of adulterants [3]. Several NPAAs, such as L-Dopa, γ-aminobutyric acid (GABA), L-theanine, and taurine offer potential health benefits when consumed in appropriate amounts. However, it is crucial to note that the safety of these amino acids can vary depending on individual intake. Excessive consumption may lead to adverse or even toxic effects. Additionally, some NPAAs, like furosine, are used to assess the nutritional quality of thermally processed foods [4].

While NPAAs play a role in plant defense, they can be harmful or toxic to humans [5]. When inadvertently incorporated into proteins, they can cause misfolding and aggregation, leading to health problems. Examples of such harmful NPAAs include homoarginine in legumes, hypoglycin A in unripe ackee fruit, L-canavanine in alfalfa, and more recently, baikiain in tara flour [6,7]. Common household methods for reducing NPAAs in legumes include soaking, rinsing, and cooking. Fermentation and processing are often employed on a larger scale, but their effectiveness can vary depending on the specific NPAA and legume.

Tara flour, a plant protein linked to adverse health effects, was implicated in 2022 outbreak of adverse effects involving Daily Harvest’s French Lentil & Leek Crumbles [7,8]. Hundreds of consumers experienced gastrointestinal distress and liver problems. The FDA identified tara flour as the likely culprit, leading to product recalls [9]. Similar issues arose with Revive Superfoods smoothies, where tara protein flour was one of the newly introduced ingredients. Research suggests that the NPAA baikiain in tara flour may be responsible since studies in mice showed that it negatively impacts liver function [7]. Hence, in the event of an outbreak of adverse effects related to food, the rapid identification and characterization of NPAAs are essential for addressing the safety of plant-based protein products.

Recently, a shift towards plant-based proteins has emerged, with various options now available to consumers. Health benefits, sustainability concerns, and the desire for animal-free alternatives drive this trend. However, the safety of such protein alternatives is largely unknown, specifically the potential for population-specific adverse health effects. These effects can vary based on genetic, dietary, and environmental factors. For example, certain populations might be more susceptible to the toxic effects of specific NPAAs due to genetic predispositions or dietary habits. Additionally, some NPAAs can interfere with nutrient absorption and metabolism, leading to health issues that might be more pronounced in certain demographic groups [10].

Early studies of NPAAs relied upon one-dimensional and two-dimensional thin-layer chromatography (TLC) [11] and paper electrophoresis [12,13] and were limited by detection limits that exceeded the normally encountered trace levels of NPAAs. High-performance liquid chromatography (HPLC) [14,15,16], gas chromatography (GC) [17,18], ion exchange chromatography [19], and capillary electrophoresis (CE) [20] are among the methods currently used for the detection of NPAAs. HPLC methods often rely upon the derivatization of the NPAAs to form a chromophore for detection by photometric detectors. The addition of mass spectrometry as a detector for HPLC allows discrimination between analytes not only by retention time but also by their mass-to-charge ratio (*m*/*z*).

To comprehensively analyze the distribution of amino acids, specifically NPAAs in various legume species, a liquid chromatography-mass spectrometry (LC-MS) method is essential. Tandem mass spectrometry (MS/MS) analysis of NPAAs and common amino acid reference standards will enable their confident identification. In comparison to traditional amino acid profiling, the proposed method of analysis offers comprehensive qualitative profiling on an array of non-proteinogenic amino acids in addition to proteinogenic amino acids. In addition, the developed method provides extended sensitivity towards NPAA and PAA identification with minimal sample volume and time of analysis. Once validated, this method can be applied to a large cohort of legume seed samples to assess quality or safety issues. Moreover, orthogonal methodologies such as gel electrophoresis and protein quantifications of total proteins in legume samples are expected to provide critical data on these macromolecules.

## 2. Materials and Methods

### 2.1. Plant Materials

**Figure 1:**
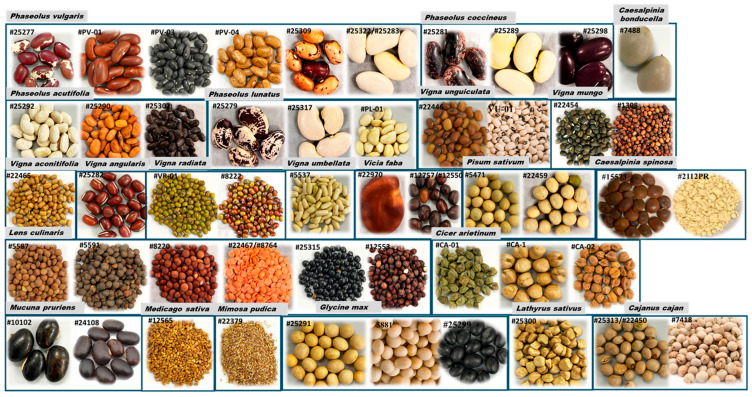
Representative seeds/beans from 22 species belonging to 13 genera.

A total of 54 dry seed samples were obtained from various sources (Table 1, Figure 1). These samples included *Phaseolus* (*P. vulgaris*, *P. lunatus*, *P. coccineus*, and *P. acutifolius*), *Lens culinaris*, *Pisum sativum*, *Cicer arietinum*, *Vigna (V. radiata*, *V. mungo*, *V. unguiculata*, *V. angularis*, *V. umbellata*, and *V. aconitifolia)*, *Vicia faba*, *Cajanus cajan*, *Lathyrus sativus*, *Mucuna pruriens*, *Mimosa pudica*, *Medicago sativa*, *Caesalpinia* (*C. spinosa* and *C. bonducella*), and *Glycine max.* In this study, whole or split seed samples were used. For each seed sample, two extraction methods for free PAAs and NPAAs were used: in the first method (refer to Method 1 below for additional details), the seed samples were soaked for 15–16 h at room temperature, and in the other extraction method (refer to Method 2 below), dried seeds were directly powdered and extracted with water. In both cases, the clear supernatant solution was analyzed in duplicate. The detailed classification of legume samples used in this study is depicted in Figure S1 based on their family, subfamily, genus, and species.

### 2.2. Chemicals and Reagents

Methanol, acetonitrile, formic acid, and ultrapure water were purchased from Fisher Chemicals (ThermoFisher, Waltham, MA, USA). The solvents used for extraction and characterization were of analytical and LC-MS grade, respectively. An AccQ-Tag Ultra derivatization kit was purchased from Waters Corporation (Milford, MA, USA). A Pierce™ Bradford Protein Assay Kit was purchased from ThermoFisher Scientific (Waltham, MA, USA), and SDS-PAGE gels and the electrophoresis system were purchased from BioRad (Hercules, California, USA). The BLUeye Pre-stained Protein Ladder was purchased from Millipore Sigma (St. Louis, MO, USA).

#### 2.2.1. Free Proteinogenic Amino Acids (PAAs)

All twenty amino acid standards (*L*-histidine, *L*-isoleucine, *L*-leucine, *L*-lysine, *L*-methionine, *L*-phenylalanine, *L*-threonine, *L*-tryptophan, *L*-valine, *L*-arginine, *L*-cysteine, *L*-glutamine, *L*-glycine, *L*-proline, *L*-tyrosine, *L*-alanine, *L*-aspartic acid, *L*-asparagine, *L*-glutamic acid, and *L*-serine) were purchased from Sigma-Aldrich (St. Louis, MO, USA).

#### 2.2.2. Free Non-Proteinogenic Amino Acids (NPAAs)

β-*N*-oxalyl-*L*-α, β-diaminopropionic acid (β-ODAP) or BOAA, 3-hydroxyproline, S-methyl-*L*-cysteine, α-aminoadipic acid, *L*-3,4-dihydroxyphenylalanine (*L*-dopa), *L*-homoserine, *L*-canavanine, azetidine-2-carboylic acid (AZE), taurine, γ-aminobutyric acid (GABA), 2,3-diaminopropionic acid (DAPA), *L*-2,4-diaminobutyric acid (DABA), *L*-theanine, *L*-citrulline, *L*-norvaline, *L*-norleucine, 5-hydroxy-*L*-tryptophan, *L*-homoserine, *N*-(2-aminoethyl) glycine (AEG), 4-amino-*L*-phenylalanine, *L*-2-amino- adipic acid, *O*-acetyl-*L*-serine, S-methyl-*L*-cysteine, cystine, taurine, 4-hydroxy-*L*-isoleucine, γ-glutamylphenylalanine, 3-hydroxy norvaline, 2,6 diaminopimelic acid (DMP), 4-amino-3-hydroxy butyric acid, *L*-mimosine, *N*^5^-methyl-*L*-glutamine, *L*-2-amino butyric acid, and *DL*-hydroxylysine were purchased from Sigma-Aldrich (St. Louis, MO, USA). Hypoglycin A was purchased from Santa Cruz Biotech Inc. (Dallas, TX, USA), 4-hydroxypipecolic acid from BOC Sciences (Shirley, NY, USA), 4-carboxy-phenylalanine from Chem-Impex Intl Inc (Wood Dale, IL, USA), γ-glutamyl-leucine from AA Blocks (San Diego, CA, USA), and γ-glutamyl-tyrosine from MedChem Express (Monmouth Junction, NJ, USA). (S)-Willardiine was purchased from ChemBlock (Hayward, CA, USA), and homoarginine, tranexamic acid, *L*-ornithine, canaline, β-cyanoalanine, ibotenic acid, and *N*-acetyl-*L*-ornithine were purchased from Cayman Chemicals (Ann Arbor, MI, USA). Pipecolic acid and 5-hydroxy pipecolic acid were purchased from 1PlusChem LLC (San Diego, CA, USA). Baikiain (4,5-dehydropipecolic acid), baikiain isomer, 3-hydroxy-methyl-phenylalanine, and 3-hydroxy methyl tyrosine were isolated at the National Center for Natural Products Research (NCNPR), University of Mississippi, University, MS, USA. The purity of each of these compounds was greater than 95%. The identity and purity of these compounds were confirmed by chromatographic and high-resolution mass spectrometry (HRMS) data.

### 2.3. Seed Extract Preparation for Soluble Protein Analysis

Twenty-two legume seed samples were ground in a Retsch MM400 mixer mill using two metal balls. One gram of the finely powdered material was resuspended in 10 mL of 1X PBS (Phosphate Buffer Saline), pH 7.2, at the concentration of 1:10 (*w*/*v*) in 50 mL round bottom tubes. The samples were vortexed and mixed overnight at 4 °C on a tube rotator. The soluble protein suspensions were then centrifuged at 12,000 rpm for 30 min at 4 °C. The supernatant containing soluble total proteins was used as a crude extract and stored at −20 °C.

### 2.4. Bradford’s Assay

The soluble protein content in the crude extracts of different legume samples was quantified using Bradford’s method [21]. The Pierce™ Bradford Protein Assay Kit was used, and protein concentrations were measured using an Ultrospec 2100 pro spectrophotometer (Amersham, England, UK). The BSA (Bovine Serum Albumin) standard curve was used for soluble protein estimation.

### 2.5. SDS-PAGE Analysis

Sodium dodecyl-sulfate polyacrylamide gel electrophoresis (SDS-PAGE) was performed according to the method described by Laemmli (1970) [22]. The 2X reducing Laemmli buffer containing 0.5 M Tris buffer–HCl (pH 6.8), glycerol, 10% SDS, bromophenol blue, and 0.01 M β-mercaptoethanol was diluted with crude extract at a 1:2 concentration. The samples were then boiled for 10 min, cooled, and centrifuged at 3000× *g* for 1 min.

For soluble protein profile analysis, 12% Criterion gels (Bio-Rad, Hercules, CA, USA) were used. A 10 µL supernatant of each protein sample was loaded onto the gel, and proteins/ peptides were separated using a Midi vertical electrophoresis unit. The BLUeye Prestained Protein Ladder served as the molecular weight standard. Electrophoresis was performed at a constant voltage of 80 V for 30 min, followed by 120 V for 45 min. The gels were stained overnight with Coomassie Brilliant Blue R-250 on a shaker and then de-stained with 40% (*v*/*v*) ethanol and 10% (*v*/*v*) acetic acid until a clear background was obtained. Finally, the gels were visualized on a GelDoc imaging system (Bio-Rad) after de-staining.

### 2.6. Sample Preparation for Free Protein and Non-Proteinogenic Amino Acids

#### 2.6.1. Preparation of Standard Solutions

A stock solution of the free proteinogenic amino acids, and the non-proteinogenic amino acids were prepared to obtain a 10 μg/mL final concentration for each analyte. The stock solution was stored at −20 °C. PAAs and NPAAs were dissolved in water.

#### 2.6.2. Seed Material and Extraction Procedure

All the seed samples selected for analysis are used for human consumption except for *M. sativa*, which is cultivated as livestock feed.

Method 1:

Cleaned seed samples of approximately 1–2 g was transferred into a 25 mL beaker, covered with twice as much water as the volume of the seeds, and soaked for about 15 h. After 15 h, the soaked water was centrifuged at 10,000 rpm, and the clear supernatant solution was used for analysis.

Method 2:

About 1 g of powdered seed sample was weighed, then sonicated in 3.5 mL of water for 30 min, followed by centrifugation for 15 min at 10,000 rpm. The supernatant was transferred to a 10 mL volumetric flask. The extraction procedure was repeated three times, and the respective supernatants were combined. The final volume was adjusted to 10 mL with water, mixed thoroughly, and centrifuged.

Finally, the AccQ-Tag Derivatization Kit from Waters (Milford, MA, USA) was applied to analyze amino acids. The clear solutions from the above two methods were mixed separately with AccQ-Tag buffer to adjust pH and AccQ-Tag derivatization reagent and heated to 55 °C to expedite the reaction rate. Briefly, a 10 μL sample was mixed with 20 μL AccQ-tag Ultra derivatization reagent and with 70 μL borate buffer (pH 8.8), mixed well, and kept in a tightly sealed vial in a heating block for 10 min at 55 °C, and similarly, a derivatization blank was prepared without sample [23]. After cooling to room temperature, 2 μL of the sample was injected.

### 2.7. Analysis Using LC-QTOF-MS/MS

The liquid chromatographic system was an Agilent Series 1290 rapid resolution (Santa Clara, CA, USA) equipped with a binary pump, and an autosampler using an Agilent Poroshell 120 Aq C18 column (150 mm × 2.1 mm, 2.7 μm particle size). The mobile phase consisted of water with 0.1% formic acid (A) and acetonitrile with 0.1% formic acid (B) at a flow rate of 0.21 mL/min. The analysis was performed using the following gradient elution: 1% B to 25% B in 25 min, then to 100% B in the next 5 min. Each run was followed by a 3 min wash with 100% B and an equilibration period of 5 min with 99% A/1% B. Two microliters of the sample were injected. The column temperature was 45 °C.

The system was coupled to a 6530 Agilent Accurate-Mass Q-TOF LC/MS (Palo Alto, CA, USA) equipped with an ESI interface. Data acquisition (2 GHz) in the profile mode was governed via MassHunter Workstation software (Agilent Technologies). The spectra were acquired in the positive ionization mode, over a mass-to-charge (*m*/*z*) range from 50 to 1000. The detection window was set to 10 ppm. An ESI source with Jet Stream technology was used with the following parameters: drying gas (N_2_) flow rate, 13 L/min; drying gas temperature, 300 °C; nebulizer pressure, 30 psig; sheath gas temperature, 300 °C; sheath gas flow, 11 L/min; capillary voltage, 3500 V; nozzle voltage, 0 V; skimmer, 60 V; Oct RF V, 750 V; and fragmentor voltage, 125 V. All the operations and the acquisition and analysis of data were controlled by Agilent MassHunter Acquisition Software Ver. A.10.1 and processed with MassHunter Qualitative Analysis software Ver. B.07.00. Accurate mass measurements were obtained employing ion correction techniques using reference masses at *m*/*z* 121.0509 (protonated purine) and 922.0098 [protonated hexakis (1H, 1H, 3H-tetrafluoropropoxy) phosphazine or HP-921] in the positive ion mode. The samples were analyzed in the all-ion MS-MS mode, where experiment 1 was carried out with a collision energy of zero and experiment 2 with a fixed collision energy of 45 eV. For the ESI-MS-MS CID experiments, precursor ions of interest were mass-selected by the quadrupole mass filter. The selected ions were then subjected to collision with nitrogen in a high-pressure collision cell. The collision energy was optimized to yield good product ion signals, which were subsequently analyzed with the ToF mass spectrometer. Analysis was performed in reflection mode with a resolving power of about 20,000 at *m*/*z* 922.0098. The instrument was set to an extended dynamic range (up to 10^5^ with lower resolving power). MS/MS spectra were recorded simultaneously at a rate of 2.0 spectra s^−1^. An isolation window of 4.0 *m*/*z* was set for the quadrupole to filter selected precursor ions for MS/MS.

### 2.8. Statistical Analysis

GraphPad Prism *v*10.4.1 was used to display the distribution of each PAA and NPAA across all 22 legume variety samples. Data were analyzed by a two-way analysis of variance (ANOVA) followed by Tukey multiple comparison testing. Results are reported in terms of the significance of the *p* value (* *p* = 0.01–0.05, ** *p* = 0.001–0.009, *** *p* = 0.0001–0.0009, and **** *p* = <0.0001).

## 3. Results and Discussions

### 3.1. Soluble Protein Analysis

Total soluble seed proteins were extracted from 38 legume samples belonging to 20 distinct species within the Fabaceae family. An SDS-PAGE analysis was conducted to profile the protein polypeptides in each species, estimate their molecular weight, and assess their relative abundances. All the tested species, except *C. bonducella*, exhibited protein bands on the SDS-PAGE gel. The absence of bands in *C. bonducella* is likely attributed to the presence of tannins, which can interfere with protein extraction, as confirmed by a negative Bradford protein assay.

The remaining 19 species displayed various protein bands ranging from 5 to 250 kDa. Each species exhibited a unique protein profile characterized by band intensity and position variation, reflecting the heterogeneity of their seed proteins [24]. However, different varieties or samples of the same species, such as *P. vulgaris*, *P. coccineus*, *V. radiata*, *V. unguiculata*, *L. culinaris*, *C. arietinum*, and *V. faba*, exhibited comparable band patterns.

**Figure 2:**
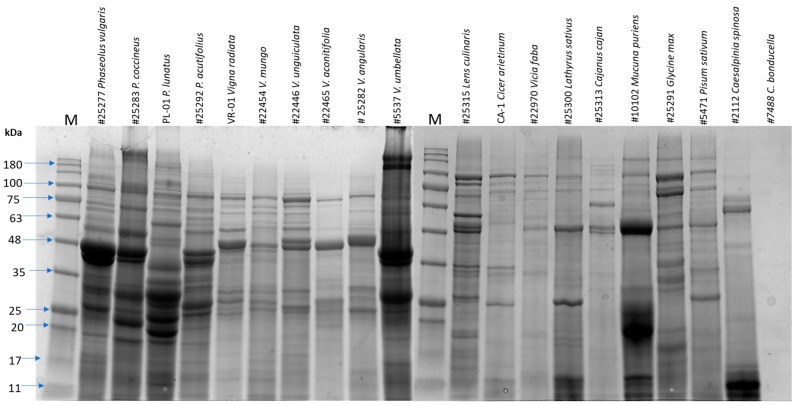
SDS-PAGE profile of crude extracts of different pulse proteins from family *Fabaceae*. ‘M’ indicates protein molecular standards, and kDa indicates the molecular weight of kilodaltons.

Globulins constitute legumes’ primary seed storage proteins, while albumins are present in smaller quantities [25]. The Bradford protein assays revealed a range of protein concentrations from 15 to 110 mg/g in the crude dry legume samples. *Phaseolus* species demonstrated the highest protein concentrations, while *V. faba* exhibited the lowest (Table 2, Figure 2). However, the small sample size and limited replication (1–3 replicates) for most samples raise concerns about the generalizability of these trends. Further investigation is needed to determine whether protein analysis varies based on seed variety, age, or geographic location.

### 3.2. Soaking

Seed extracts and powdered extracts showed similar patterns of free protein and non-proteinogenic amino acids, although the powdered samples generally displayed higher peak intensities. Soaking was found to reduce the levels of free protein and non-proteinogenic amino acids compared to the original seeds. About 1–2 g of the sample was added to a beaker containing 5 mL of HPLC grade water. The samples were soaked for approximately 15 h at room temperature then placed on filter paper to eliminate surface moisture. These soaked samples were weighed to ±0.0001 g to determine moisture gain. The samples were dried in an oven at 70 °C for 24 h, cooled to room temperature, and weighed to obtain a post-soaking weight.

**Figure 3:**
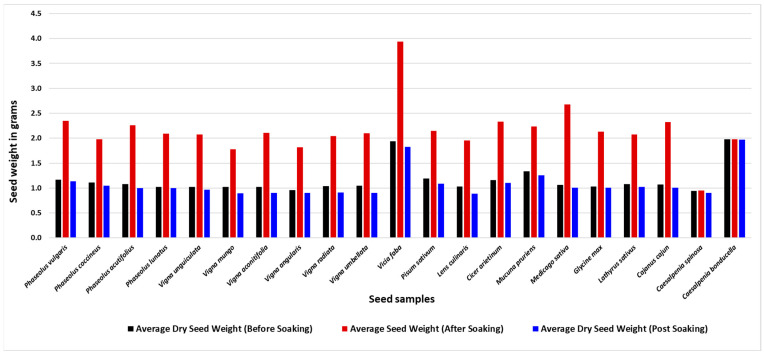
The average weight of seeds before soaking, after soaking, and dried post-soaking of 21 samples after a soaking time of 15 h.

Soaking is a common preparation method for legumes, facilitating the extraction and elimination of certain NPAAs as well as enabling a reduced cooking time [26]. Our analysis revealed that most legumes exhibit significant weight gain due to water absorption during soaking, indicating effective hydration. However, *Caesalpinia* species showed no change in weight. Despite this, most legumes experienced a notable reduction in dry weight after soaking, suggesting that soluble compounds were extracted. These findings confirm the efficacy of water extraction for obtaining PAAs and NPAAs from legumes and support the conventional practice of soaking them before cooking. Compared to the un-soaked seeds, the soaked seed samples were larger and around double in weight. The dried soaked seed weights were close to the original seed weight. The differences between the dry weight of the pre-soaked, soaked, and post-soaking dry seeds are depicted in Figure 3.

To determine the qualitative differences in the free proteinogenic and non-proteinogenic amino acid profiles from edible seeds, the seeds (Table 1) were subjected to whole seed soaking (Method 1 under Section 2.6.2), with the dried seeds then ground into fine powder and subjected to an exhaustive extraction method (Method 2 under Section 2.6.2) using water as the solvent. Upon comparison there is no qualitative difference observed in the proteinogenic and non-proteinogenic amino acids profiles. We considered the optimal peak intensities of analytes observed in the samples obtained through Method 1 in comparison to Method 2, for the further derivatization of samples using AccQ. Tag reagent was used to generate fluorescent labelled amino acids with greater sensitivity in terms of detection via mass spectrometric detectors coupled with liquid chromatography. Although grinding un-soaked seed samples produced adequate peak intensities, to maintain detection sensitivity, minimize saturation, and sustain repeatability in retention times, the soaked seed samples were chosen to represent qualitative profiles.

Non-proteinogenic amino acids have been studied to a lesser extent than PAAs but have been shown to have an important role in the quality and safety of food. The chemical and physical diversities of metabolites make them difficult to identify based solely on MS data. In the present study, the analyte identification in seed samples was achieved primarily through an accurate mass-based search for molecular formula followed by a search of published literature search engines (Google, PubMed) and databases (SciFinder). Reference compounds of these putative identifications were then subjected to a tandem MS/MS experiment side-by-side with the sample. By comparing the MS/MS spectra and retention times of the reference compounds with the molecules of interest in the sample, the identities of the molecules were confirmed.

A sensitive LC–QToF–MS system was employed to analyze the free form of PAAs and NPAAs that are expected to be at relatively low concentrations in legume samples. Direct analysis (un-derivatized) and derivatization approaches were used for the analysis of free amino acids. Direct analysis is rapid and simple but was found to be less sensitive due to matrix complexity and interferences. To increase the sensitivity and selectivity of amino acid analysis in a complex matrix, the derivatization approach was used to improve chromatographic separation. A readily available 6-aminoquinolyl-*N*-hydroxysuccinimidyl carbamate (AQC) reagent was utilized to effectively derivatize the amino acids and enhance the selectivity and sensitivity. Both primary and secondary amines are reported to form stable urea adducts. Excess AQC reagent reacts with water to produce 6-aminoquinoline (AMQ), which is chromatographically resolved or further reacts with another AQC molecule to form a stable bis-aminoquinoline urea [23]. The resulting undesired side products do not often interfere with separating and identifying analytes of interest.

Upon the derivatization of both extracts with AQC, the derivatized amino acids (PAAs and NPAAs) were separated by liquid chromatography and detected by mass-specific detection using [M+H]^+^ ions in the positive ion mode with extractive ion monitoring (EIM). This method detected and confirmed mono-derivatized and bis-derivatized AQC-amino acid adducts. Most amino acids produced a mono-derivatized product with AQC, while cystine, lysine, 2, 3 diamino propionic acid, 2, 4-aminobutyric acid, *L*-ornithine, AEG, β-methylamino-*L*-alanine (*L*-BMAA), DMP, *L*-canaline, and hydroxylysine were bis-derivatized. A similar phenomenon has been reported in skin-related amino acids analysis [27]. The major advantage of this method is that no sample solvent evaporation is necessary. The samples were prepared at a 1:10 dilution in the reagents, which may have hindered the identification of low-abundance compounds.

### 3.3. Fragmentation Patterns PAAs and NPAAs Using LC-QToF-MS Analysis

This study examined fragmentation patterns of protonated amino acids using LC-QToF-MS/MS with collision-induced dissociation (CID) in the mass range of 50 to 1100 Da. The fragmentation patterns of PAAs and NPAAs were analyzed, excluding glycine (75 Da) and alanine (89 Da). For each derivatized amino acid, fragmentation at the 6-aminoquinoline carbonyl group produced a high-intensity common fragment ion at *m*/*z* 171.0553, along with 6-aminoquinoline (AMQ, *m*/*z* 145.076 C_9_H_8_N_2_), and a fragment corresponding to the amino acid’s original mass. The observed fragment ions of protonated amino acids are summarized in Table 3.

Derivatized amino acids showed predominantly the fragment ions (*m*/*z* 171.0553, 145.0760) associated with the derivatization agent while also showing ions similar to those seen with underivatized or native amino acid fragment ions, i.e., fragment ions of the amino acid backbone (Table 3). Amino acids containing sulfur, such as methionine and cysteine, exhibited losses of ammonia (17 Da, NH_3_), water (18 Da, H_2_O), carbon monoxide (CO), ethanimine (C_2_H_5_N), and methyl thiol (CH_3_SH). Branched-chain amino acids or hydrophobic compounds like leucine, isoleucine, and valine showed sequential losses of H_2_O, CO, and NH_3_. Similarly, protonated aromatic amino acids, such as tryptophan, phenylalanine, and tyrosine, demonstrated losses of CH_2_CO, CO_2_, NH_3_, H2O+CO, HCN, CH_3_, H_2_O, and CO. Polar uncharged amino acids, including glutamine, asparagine, threonine, and serine, showed losses of NH_3_, H_2_O+CO, and H_2_O. The observed fragment losses for the negatively charged side chains, such as those in glutamic and aspartic acid, included H_2_O and H_2_O+CO. Positively charged amino acids, including histidine, arginine, lysine, and ornithine, showed losses of H_2_O+CO, NH_3_, CO_2_, or combinations thereof. While most amino acids fragmented uniquely, some structurally similar amino acids produced common fragment ions [28,29]. Similar patterns of fragmentation were observed for NPAAs (Table 3). Extracted ion chromatograms (EIC) for each analyte with mass spectrometry information in MS and MS/MS modes for free PAAs and NPAAs are presented in Figure S2.

### 3.4. Proteinogenic Amino Acids (PAAs) Content

**Figure 4:**
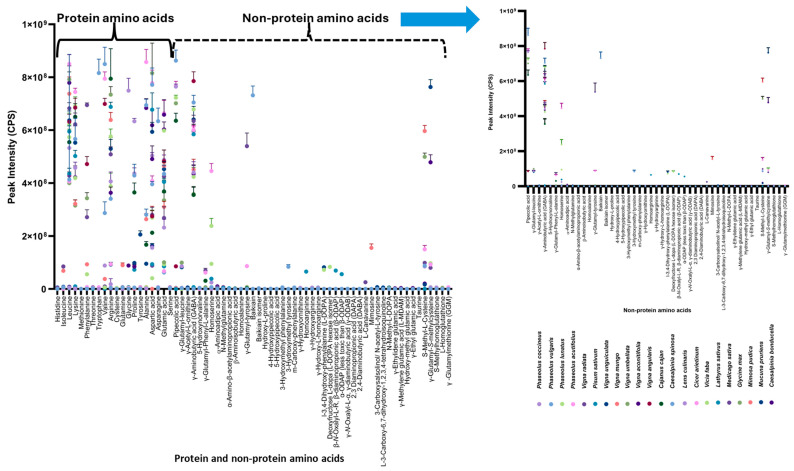
Relative distribution of free proteinogenic and non-proteinogenic amino acids across different Leguminosae seeds in water under soaking conditions.

The 20 free proteinogenic amino acids were detected in most seed and seed powder samples (Table 4 and Figure 4). The PAAs mostly found in these samples were leucine, lysine, alanine, aspartic acid, and glutamic acid. The analysis of *P. sativum*, *L. culinaris*, *C. arietinum*, and *V. faba* analysis revealed lysine, proline, leucine, and arginine as major common amino acids with lower levels of methionine, cysteine, histidine, and tryptophan. Methionine and tryptophan were identified at minimal levels in lentils.

Similarly, *M. pruriens* showed arginine, isoleucine, leucine, lysine, and tyrosine as major PAAs, whereas methionine, cystine, tryptophan, and histidine were present in lower amounts. The *P. coccineus* analysis showed proline, glycine, and valine as major compounds. *V. unguiculata* and *V. aconitifolia* were rich in alanine, arginine, and proline. *V. umbellata*, *M. sativa*, and *P. lunatus* showed arginine, leucine, lysine, and phenylalanine as major PAAs. The samples of *P. acutifolius* and *P. vulgaris* showed alanine, lysine, valine, and leucine as major compounds with lower amounts of methionine, histidine, cysteine, and tyrosine. *V. angularis* was high in lysine, leucine, phenylalanine, and valine, whereas lysine, valine, isoleucine, leucine, phenylalanine, and alanine contents were high in *V. radiata* and *V. mungo*. *L. sativus* showed higher valine, leucine, lysine, proline, and serine levels.

In the *C. bonducella* powder samples, the major compounds were aspartic acid, glycine, leucine, histidine, and isoleucine, but at lower amounts were methionine, tryptophan, phenylalanine, cysteine, and valine. Glutamic and aspartic acids were major components in all samples except in *C. cajan*, where alanine has relatively higher levels of lysine, valine, leucine, and glutamine. In most legume samples, the sulfur-containing amino acids methionine and cysteine were the limiting essential amino acids [30]. In tara powder from *C. spinosa*, lysine is the limiting amino acid, with leucine, arginine, glycine, and alanine as major ones. Asparagine is the most important PAA used in the biosynthesis of proteins and was found in all the samples.

The amino acids in the seed extracts showed more variability than they did in the powder extracts. It is plausible that the extractability of such PAAs may be more efficient with powdered samples compared to intact, soaked legume seeds. While most PAAs were not detected, trace amounts of phenylalanine, valine, alanine, glutamic acid, and serine were found. Notably, *C. spinosa* extracts exhibited higher amino acid levels than *C. bonducella*. No significant weight changes were observed in soaked seeds of either *Caesalpinia* species.

### 3.5. Free Non-Proteinogenic Amino Acid (NPAAs) Content

NPAAs are primarily found in free form, but some can be conjugated to carbohydrates or linked to glutamyl residues [31]. Many NPAAs have structural similarities to PAAs and often contain hydroxylated aromatic or heterocyclic rings. Heterocyclic NPAAs may include oxygen, nitrogen, and sulfur atoms in their structures [32]. These compounds are generally considered more toxic than PAAs and can deter herbivores through direct toxicity or by making plants unpalatable. Combinations of NPAAs often have a stronger deterrent effect than individual compounds [33]. A previous study observed changes in NPAA levels during the germination of legume seeds [34].

PAAs and NPAAs do contribute taste and flavor to food products especially in terms of being savory or bitter. These factors vary based on chemical composition along with protein breakdown during digestion as well as the concentrations of PAAs and NPAAs. Specific NPAAs may have an impact on taste and flavor profiles [35]. According to Kim et al., proline, asparagine, tyrosine, alanine, leucine, and glutamate can contribute to the bitterness of food products. In addition, the hydrophobic and electronic properties of amino acids and the critical spatial structure of peptides influence the intensity of bitterness [36]. Traditional processing like soaking before cooking increases the nutritional value and lowers the amount of harmful components and antinutrients, such as NPAAs, phytic acid, tannins, lectin, and trypsin inhibitor. Considering the above factors, it is evident that the analyzed and identified PAAs and NPAAs likely impact the taste and flavor profiles of respective legume seeds, but, because of the complexities in PAAs and NPAAs composition, investigations to establish specificity in taste related to non-proteinogenic amino acids will be challenging.

Our analysis revealed qualitative differences in NPAAs among the seeds. Several diverse chemical structures derived from nicotinic acid or common proteinogenic amino acids were detected in these seeds and could serve as chemotaxonomic markers. Trigonelline (a nicotinic acid derivative) and γ-aminobutyric acid (GABA) were detected in all the seeds. Some of the NPAAs, such as α-aminoadipic acid, homoserine, pipecolic acid, hydroxy norvaline, acetyl ornithine, *O*-oxalyl homoserine, hydroxy arginine, α-ODAP_,_ or L-dopa, were detected in these samples with variable concentrations and are dependent upon plant species. While their effects on humans are largely unknown, toxicity cannot be ruled out. For instance, *L*-canavanine in *M. sativa* is toxic to herbivores and interferes with protein synthesis [37]. Lathyrism, a neurodegenerative disease, can be caused by the excessive consumption of *L. sativus* seeds due to the presence of β-ODAP [38]. Conversely, *L*-dopa (*L*-3,4-dihydroxyphenylalanine), extracted from *M. pruriens* seeds, is claimed to treat Parkinson’s disease [39].

As reported earlier [40,41], our analysis revealed distinct chemical profiles among the examined *Vigna* and other Faboideae species. Notably, *Vigna* species were characterized by the absence of pipecolic acid, a compound found in other genera. Furthermore, we observed significant differences in the types and concentrations of NPAAs between *Vicia* and *Lathyrus* seeds. *Vicia* species preferentially accumulated C6 guanidino amino acids, such as arginine and γ-hydroxy arginine, while *Lathyrus* species contained higher levels of C7 guanidino amino acids, including homoarginine, γ-hydroxy homoarginine, and lathyrine. These unique chemical markers provide a reliable method for distinguishing between species within these genera.

The analysis of *M. pruriens* revealed the presence of several non-proteinogenic amino/imino acids, including *L*-dopa, *L*-3-carboxy-6,7-dihydroxy-1,2,3,4-tetrahydroisoquinoline, and 1-methyl-3-carboxy-6,7-dihydroxy-1,2,3,4-tetrahydro-isoquinoline. A more diverse NPAA profile was observed with *L. culinaris* varieties [42], where detected components included γ-hydroxyarginine (γ-OH-Arg), γ-hydroxyornithine (γ-OH-Orn), homoarginine (Har), γ-hydroxy-*L*-homoarginine, γ-acetylornithine, γ-glutamyl-phenylalanine, homoserine, methylglutamine, and γ-glutamyl leucine. Several other NPAAs, such as γ-hydroxynorvaline, δ-hydroxynorvaline, homoserine, and δ-acetylornithine, were found in varying distributions.

The analysis of *G. max* seeds resulted in the identification of eight NPAAs including GABA, γ-glutamyl-phenyl-L-alanine, γ-glutamyl tyrosine, hydroxy-L-proline, 5-methyl-L-cysteine, and γ-glutamyl-*S*-methyl-L-cysteine (GMC). These are dipeptides belonging to the family of N-acyl-α-amino acids and derivatives. According to Shibata et al., these dipeptides impact the taste of food products such as Kokumi [43]. Additionally, two other NPAAs were identified along with dipeptides, i.e., pipecolic acid and α-aminoadipic acid in very low amounts.

**Figure 5:**
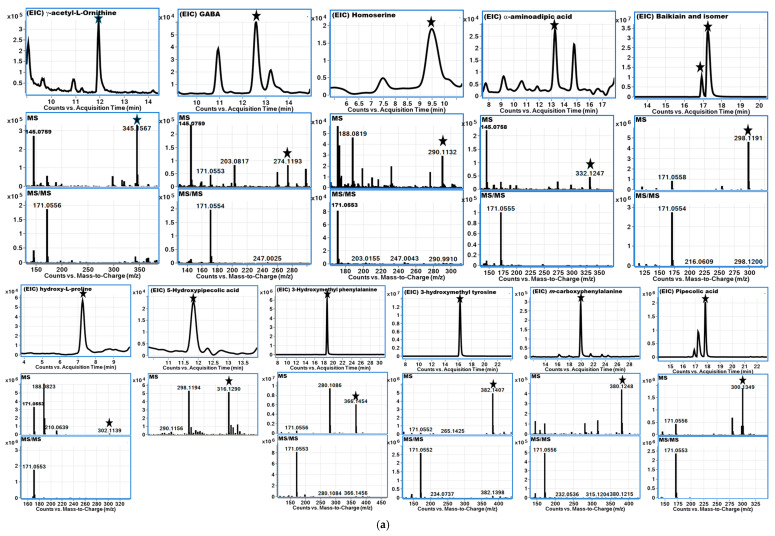

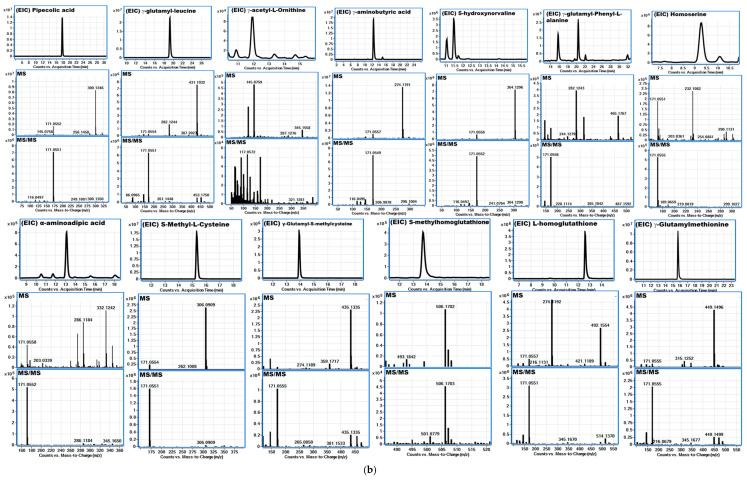
Extracted ion chromatograms (EIC) for each analyte with mass spectrometry information in MS and MS-MS modes of (**a**) *C. spinosa* (tara) seed powder samples; (**b**) *P. vulgaris* (common bean) soaked seed samples.

In the *Caesalpinioideae* subfamily, *C. bonducella* seeds contained seven NPAAs, including γ-ethylidene glutamic acid, γ-aminobutyric acid, γ-ethylidene glutamic acid, γ-methylene glutamic acid, hydroxy-methyl glutamic acid, and γ-ethyl glutamic acid. *C. spinosa* seeds, on the other hand, contained 13 NPAAs, including γ-aminobutyric acid (GABA), homoserine, baikiain, baikiain isomer, pipecolic acid (PA), 3-hydroxyproline, 5-hydroxypipecolic acid (5-HPA), 4-hydroxypipecolic acid (4-HPA), diaminopimelic acid (DAP), 3-hydroxymethyl phenylalanine (3-HMP), 3-hydroxymethyl tyrosine (3-HMT), *meta*-carboxyphenylalanine, acetylornithine, and α-aminoadipic acid (Table 4, Figure 5a). Several compounds, including baikiain, 3-HMP, 3-HMT, 5-HPA, 4-HPA, and PA, have been previously reported in *C. spinosa* [44]. Finally, *M. pudica* seeds contained six NPAAs, including GABA, α-aminoadipic acid, mimosine, γ-glutamyl-leucine, γ-glutamyl-3-phenyl-*L*-alanine, and pipecolic acid as shown in Table 4.

### 3.6. Non-Proteinogenic Sulfur Amino Acids

The analysis of seeds of 21 species across different genera within the *Fabaceae* family revealed a broad distribution and accumulation of *S*-methyl-*L*-cysteine and its γ-glutamyl derivative. *S*-Methyl-*L*-cysteine was detected in all the examined *Phaseolus* and *Vigna* species, as well as in *C. cajan*, *L. culinaris*, *C. arietinum*, and *G. max*. Within the genus *Vigna*, *S*-methyl-*L*-cysteine distribution aligns with phylogenetic groups [40].

*V. radiata* and *P. vulgaris* represent closely related members of the *Phaseoleae* tribe within the *Faboideae* family, followed by *G. max* and *C. cajan* [45]. *S*-Methyl-*L*-cysteine and γ-glutamyl-*S*-methyl-cysteine (GMC) were detected in *V. radiata*, *V. unguiculata*, *V. umbellata*, and *V. aconitifolia*, but only trace amounts were observed in *V. mungo*, *V. angularis*, *G. max*, *C. cajan*, and *L. culinaris.* Conversely, γ-glutamyl methionine was detected in major amounts in *V. mungo* and trace amounts in *C. cajan*, but not detected in *V. angularis* and *V. radiata.*

*S*-Methyl-homoglutathione was detected in *V. radiata.*, *V. umbellata*, and *V. aconitifolia*, but in low amounts in *V. mungo*, and not detected in *V. angularis* and *V. unguiculata.* Similarly, L-homoglutathione was detected in *V. radiata.*, *V. umbellata*, *V. aconitifolia*, and *V. angularis*, but in low amounts in *V. mungo* and not detected in *V. unguiculata.* These compounds were not detected in *M. pruriens*, *M. sativa*, *M. pudica*, *C. spinosa*, and *C. bonducella* (Table 4).

All the samples contained γ-aminobutyric acid, but *C. cajan* exhibited lower concentrations of α/β/γ-aminobutyric acid than γ-aminobutyric acid alone. Low levels of α-aminobutyric acid have also been identified in *Phaseolus* species (Figure 5b). Further, the NPAAs identified in *P. vulgaris* are represented in Figure 5b with notable amounts of pipecolic acid, GABA, and S-methyl-L-cysteine. Bell [26] reported that *Phaseolus* species accumulate significantly higher concentrations of GMC than *Vigna* spp. The common bean (*P. vulgaris*) is known to store sulfur-containing NPAAs, such as GMC [46,47], *S*-methyl-*L*-cysteine [48,49,50], and *S*-methyl-homoglutathione [51]. Several grain legumes, including *Phaseolus* and *Vigna* species, store sulfur in the form of NPAAs within their seeds, demonstrating that in *P. vulgaris*, free *S*-methyl cysteine levels are elevated during early seed development, subsequently declining as GMC accumulates [52].

Four species of *Phaseolus*, *L. culinaris* and two *Vigna* species were identified to contain both *S*-methyl cysteine and GMC in their seeds. The *Phaseolus*, *Lens*, and *Vigna* genera encompass species that are important for human consumption, including the common bean (*P. vulgaris*), mung bean (*V. radiata*), and cowpea (*V. unguiculata*). *S-*Methyl-homoglutathione was detected in mature seeds of *V. radiata* and *P. vulgaris* [46,51]. *S-*Methyl-homoglutathione was observed in *Phaseolus*, *L. culinaris*, and *Vigna* species.

Forty-seven free NPAAs and twenty free PAAs were identified across various legume samples in this study. Most NPAAs (26/45) were definitively identified based on accurate mass spectrum, retention time, and MS-MS fragment ions. While reference standards were unavailable for the remaining 21 NPAAs, they were tentatively identified based on accurate mass spectra and fragment ions. Most samples contained all 20 PAAs. All 22 species showed the presence of GABA and α-aminoadipic acid. Nineteen of twenty-two species contained γ-glutamyl-phenyl-*L*-alanine, while eighteen contained pipecolic acid, and sixteen contained γ-glutamyl-leucine and *S*-methyl-*L*-cysteine. Fifteen samples showed the presence of γ-acetyl-*L*-ornithine. Fourteen contained γ-glutamyl-*S*-methyl cysteine, and nine contained homoserine. Other compounds were detected in fewer samples (Table 4).

With amino acids playing a crucial role in protein synthesis and metabolic homeostasis, amino acid profiling has become a valuable tool for identifying and monitoring primary metabolic disorders. These disorders may manifest biochemically as metabolic acidosis, hyperammonemia, hypoglycemia (with appropriate or increased ketosis), multi-system dysfunction, developmental delay, encephalopathy, coma, or death [53].

### 3.7. Statistical Analysis

The statistical analyses used to determine the distribution of proteinogenic and non-proteinogenic amino acids in the soaked bean samples include two-way ANOVA along with a Tukey multiple comparison testing analysis. It is observed that the “*p*” value is <0.05 for different free proteinogenic and non-proteinogenic amino acids in legume seeds, which were listed under Table 1. This defines the significance of the results on proteinogenic and non-proteinogenic amino acids in the edible soaked seeds of the Leguminosae family.

## 4. Conclusions

Legumes are a major protein source in many cultures and offer a rich supply of non-animal protein for the future. Twenty free proteinogenic amino acids (PAAs) form the building blocks of proteins, while free non-proteinogenic amino acids (NPAAs) have diverse roles in physiology, ranging from beneficial to harmful. The distribution of NPAAs varies among legume species, often serving species-specific or unique marker compounds. A liquid chromatography quadrupole time-of-flight high-resolution mass spectrometry (LC-QToF-MS) method was developed to simultaneously analyze free proteinogenic and non-proteinogenic amino acids as their respective 6-aminoquinolyl-*N*-hydroxysuccinimidyl carbamate (AQC) derivatives in 22 legume (Fabaceae) seeds. The distribution of PAAs and NPAAs was determined across 13 genera (*Phaseolus*, *Vigna*, *Lens*, *Pisum*, *Lathyrus*, *Mimosa*, *Medicago*, *Vicia*, *Cajanus*, *Caesalpinia*, *Cicer*, *Mucuna*, and *Glycine*) based on retention time indices, accurate mass, and fragmentation ion matching in comparison with respective reference standards. Seeds of legumes showed a diverse range of non-proteinogenic amino acids with different chemical and biochemical properties. At least 7–14 NPAAs were identified in each seed sample, with 47 NPAAs and 20 PAAs detected. Most were newly identified, especially the sulfur-containing non-proteinogenic amino acids. Despite the benefits or nutritional properties, toxicities with non-proteinogenic amino acids may sometimes pose a risk to humans or livestock. All the seeds analyzed in this study showed different absorption rates and quantities of water absorbed, which are important quality parameters useful for many industrial applications. The Bradford assay provided the soluble protein concentration, whereas the standard SDS-PAGE method was employed to resolve the protein polypeptides within proteomic mixtures. The resulting method is expected to aid in the qualitative and quantitative analysis of commercial plant-based protein products and could serve as a valuable tool for the rapid dereplication of NPAA-based causative agents if specific protein-based products are implicated in adverse effects. Furthermore, this analytical technique allows for the frequent monitoring and analysis of toxic compounds to help in the prevention or reduction of exposure. Ultimately, this work will contribute to a more comprehensive understanding of legume amino acids (PAAs and NPAAs) composition and its biological effects.

Considering the minimal regulatory standards for plant-based food products, it is imperative to address the safety profile of food products, especially those which contain edible beans or seeds. The findings of this study highlight the need for analyzing NPAAs in food products since they plausibly cause metabolic changes, and may elicit toxicity in humans.

## Figures and Tables

**Table 1:** Code #, scientific name, common name, and source information of seeds from different Genera.

#	NCNPR Code #	Plant Name	Common Name	Plant Part	Source Information
1	25277	*Phaseolus vulgaris*	*Anasazi bean*	Seed	Missouri Botanical Garden, St. Louis, MO, USA
2	PV-01	*Phaseolus vulgaris*	Kidney bean	Seed	Local store
3	PV-03	*Phaseolus vulgaris*	Black turtle bean	Seed	Amazon.com
4	PV-04	*Phaseolus vulgaris*	Pinto bean	Seed	Local store
5	25309	*Phaseolus vulgaris*	Cranberry bean	Seed	Missouri Botanical Garden, St. Louis, MO, USA
6	25322	*Phaseolus vulgaris*	*Cannellini* bean	Seed	
7	25283	*Phaseolus coccineus*	Black Runner/Ayocote Pinto	Seed	
8	25281	*Phaseolus coccineus*	Scarlet Runner	Seed	
9	25289	*Phaseolus coccineus*	Corona bean	Seed	
10	25298	*Phaseolus coccineus*	Ayocote Morado bean	Seed	
11	25292	*Phaseolus acutifolius*	White tepary bean	Seed	
12	25290	*Phaseolus acutifolius*	Brown tepary bean	Seed	
13	25302	*Phaseolus acutifolius*	Black tepary bean	Seed	
14	25279	*Phaseolus lunatus*	Christmas speckled lima bean	Seed	
15	25317	*Phaseolus lunatus*	Large lima bean	Seed	
16	PL-01	*Phaseolus lunatus*	Lima bean	Seed	Local store
17	22446	*Vigna unguiculata*	Black-eyed pea or cowpea	Seed	Missouri Botanical Garden, St. Louis, MO, USA
18	VU-01	*Vigna unguiculata*		Seed	Medicinal Plant Garden, Oxford, MS
19	22454	*Vigna mungo*	Urdbean or black gram	Seed	Missouri Botanical Garden, St. Louis, MO, USA
20	1308	*Vigna mungo*		Seed	Local store
21	22465	*Vigna aconitifolia*	Mat bean, moth bean, matki, or dew bean	Seed	Missouri Botanical Garden, St. Louis, MO, USA
22	25282	*Vigna angularis*	Adzuki bean azuki bean, aduki bean, red bean, or red mung bean	Seed	
23	VR-01	*Vigna radiata*	Green gram or moong bean	Seed	Local store
24	8222	*Vigna radiata*		Seed	
25	5537	*Vigna umbellata* previously *Phaseolus calcaratus*	Ricebean or rice bean	Seed	
26	22970	*Vicia faba*	Broad bean, fava bean, or faba bean	Seed	Missouri Botanical Garden, St. Louis, MO, USA
27	12757	*Vicia faba*		Seed	
28	12550	*Vicia faba*		Seed	
29	5471	*Pisum sativum*	Pea	Seed	Local store
30	22459	*Pisum sativum*		Seed	Missouri Botanical Garden, St. Louis, MO, USA
31	5587	*Lens culinaris*	Brown lentil	Seed	Local store
32	5591	*Lens culinaris*	Dark brown lentil	Seed	
33	8220	*Lens culinaris*	Brown lentil	Seed	Commercial source
34	8764	*Lens culinaris*	Masoor lentil or red lentil	Seed	Missouri Botanical Garden, St. Louis, MO, USA
35	22467	*Lens culinaris*	Masoor lentil or red lentil	Seed	
36	25315	*Lens culinaris*	Beluga: black, bead-like lentil	Seed	
37	12553	*Lens culinaris*	Dark brown lentil	Seed	
38	CA-1	*Cicer arietinum*	Chickpea or garbanzo bean	Seed	Local store
39	CA-01	*Cicer arietinum*	Green chickpea	Seed	
40	CA-02	*Cicer arietinum*	Brown chickpea	Seed	
41	10102	*Mucuna pruriens*	Monkey tamarind, velvet bean	Seed	Commercial source
42	24108	*Mucuna pruriens*		Seed	Missouri Botanical Garden, St. Louis, MO, USA
43	12565	*Medicago sativa*	Alfalfa	Seed	
44	22379	*Mimosa pudica*	Lajwanti seeds, Chui Mui seeds	Seed powder *	
45	25291	*Glycine max*	Golden soybean, soybean, or soya bean	Seed	
46	5881	*Glycine max*		Seed	
47	25299	*Glycine max*	Black soybean	Seed	
48	25300	*Lathyrus sativus*	Grass pea, cicerchia, blue sweet pea, chickling pea, chickling vetch, Indian pea, white pea, and white vetch	Seed	
49	25313	*Cajanus cajan*	Pigeon pea	Seed	
50	7418	*Cajanus cajan*		Seed	
51	22450	*Cajanus cajan*		Seed	
52	15573	*Caesalpinia spinosa*	Tara (Quechua), Peruvian carob, or spiny holdback-	Seed	
53	2112PR	*Caesalpinia spinosa*		Seed powder *	Commercial source
54	7488	*Caesalpinia bonducella*	Grey nicker, nicker bean, fever nut or knicker nut, or ivy gourd	Seed	

* indicates the sample is in powder form.

**Table 2:** The soluble protein concentrations in different bean samples were estimated using Bradford assay. The protein concentrations vary in different samples, and no protein was obtained from *C. bonducella*, in congruence with SDS-PAGE analysis.

#	NCNPR #	Species	Soluble Protein Concentration (mg/g Dry Weight)
1	25277	*Phaseolus vulgaris*	110
2	25283	*Phaseolus coccineus*	87
3	PL-01	*Phaseolus lunatus*	111
4	25292	*Phaseolus acutifolius*	69
5	VR-01	*Vigna radiata*	45
6	22454	*Vigna mungo*	39
7	22446	*Vigna unguiculata*	31
8	22465	*Vigna aconitifolia*	29
9	25282	*Vigna angularis*	23
10	5537	*Vigna umbellata*	71
11	25315	*Lens culinaris*	57
12	CA-1	*Cicer arietinum*	21
13	22970	*Vicia faba*	15
14	25300	*Lathyrus sativus*	30
15	25313	*Cajanus cajan*	26
16	10102	*Mucuna puriens*	98
17	25291	*Glycine max*	94
18	5471	*Pisum sativum*	52
19	2112PR	*Caesalpinia spinosa*	45
20	7488	*Caesalpinia bonducella*	-

Note: *Medicago sativa* and *Mimosa pudica* samples not analyzed due to presence of excess mucilage.

**Table 3:** Extracted high-resolution masses (*m*/*z*) of mono-derivatized and bis-derivatized charged ions of AQC-derivatized pure amino acids.

#	Compound Name	Chemical Formula	Rt (min)	Mass	[M+H]^+^	AQC Tag Mass	Protonated Fragment Ions (*m*/*z*) of Native Compounds
1	Histidine (H)	C_6_H_9_N_3_O_2_	7.16	155.0695	156.0768	326.1251 (326.1248) **	110.0711 [M+H−H_2_O−CO]^+^, 93.0446 [M+H−H_2_O−CO−NH_3_]^+^
2	Isoleucine (I)	C_6_H_13_NO_2_	21.87	131.0946	132.1019	302.1504 (302.1499)	86.0964 [M+H−H_2_O−CO]^+^, 69.0699 [M+H−H_2_O−CO−NH_3_]^+^
3	Leucine (L)	C_6_H_13_NO_2_	22.34	131.0946	132.1019	302.1503 (302.1499)	86.0961 [M+H−H_2_O−CO]^+^
4	Lysine (K)	C_6_H_14_N_2_O_2_	16.45	146.1055	147.1128	487.2091 * (487.2088)	130.0861 [M+H−NH_3_]^+^, 84.0806 [M+H−NH_3_−H_2_O−CO]^+^
5	Methionine (M)	C_5_H_11_NO_2_S	17.92	149.051	150.0583	320.1071 (320.1063)	133.0315 [M+H−NH_3_]^+^, 104.05275 [M+H−H_2_O−CO]^+^
6	Phenylalanine (F)	C_9_H_11_NO_2_	23.39	165.079	166.0863	336.1348 (336.1343)	120.0806 [M+H−H_2_O−CO]^+^, 103.0541 [M+H−H_2_O−CO−NH_3_]^+^, 93.0698, 91.0541
7	Threonine (T)	C_4_H_9_NO_3_	11.33	119.0582	120.0655	290.1141 (290.1135)	102.0549 [M+H−H_2_O]^+^, 74.0600 [M+H−H_2_O−CO]^+^
8	Tryptophan (W)	C_11_H_12_N_2_O_2_	24.35	204.0899	205.0972	375.1460 (375.1452)	188.0703 [M+H−NH_3_]^+^, 170.05975 [M+H−NH_3_−H_2_O]^+^, 146.0597 [M+H−CH_2_CO]^+^, 143.0727 [M+H−NH_3_−H_2_O−CO_2_]^+^, 118.0649 [M+H−CH_2_CO−CO]^+^, 91.0541 [M+H−CH_2_CO−CO−HCN]^+^
9	Valine (V)	C_5_H_11_NO_2_	18.15	117.079	118.0863	288.1349 (288.1343)	72.0808 [M+H−H_2_O−CO]^+^, 55.0543 [M+H−H_2_O−CO−NH_3_]^+^
10	Arginine (R)	C_6_H_14_N_4_O_2_	8.39	174.1117	175.119	345.1679 (345.167)	116.0704 [M+H−CH_5_N_3_ (guanidine group)]^+^ 70.0655 [M+H−CH_5_N_3_−H_2_O−CO]^+^
11	Cysteine (C)	C_3_H_7_NO_2_S	13.88	121.0197	122.027	292.0754 (292.0750)	88.0392 [M+H−H_2_S]^+^(minor), 76.0215 [M+H−H_2_O−CO]^+^ 58.9952 [M+H−H_2_O−CO−NH_3_]^+^
12	Glutamine (Q)	C_5_H_10_N_2_O_3_	9.02	146.0691	147.0764	317.1249 (317.1244)	130.0497 [M+H−NH_3_]^+^, 101.0549 [M+H−H_2_O−CO]^+^, 84.0443 [M+H−NH_3_−H_2_O−CO]^+^, 56.0497 [M+H−NH_3_−H_2_O−2CO]^+^
13	Glycine (G)	C_2_H_5_NO_2_	9.61	75.032	76.0393	246.0875 (246.0873)	−
14	Proline (P)	C_5_H_9_NO_2_	13.36	115.0633	116.0706	286.1191 (286.1186)	70.0651 [M+H−H_2_O−CO]^+^
15	Tyrosine (Y)	C_9_H_11_NO_3_	17.61	181.0739	182.0812	352.1300 (352.1292)	165.0543 [M+H−NH_3_]^+^, 147.0439 [M+H−NH_3_−H_2_O]^+^, 136.0754 [M+H−H_2_O−CO]^+^, 123.0439 [M+H−NH_3_−H_2_O−CH_2_CO]^+^, 119.0491 [M+H−H_2_O−CO−NH_3_]^+^, 95.0490 [M+H−NH_3_−H_2_O−CH_2_CO−CO]^+^
16	Alanine (A)	C_3_H_7_NO_2_	12.22	89.0477	90.055	260.1036 (260.1030)	−
17	Aspartic acid (D)	C_4_H_7_NO_4_	10.29	133.0375	134.0448	304.0928 (304.0928)	116.0340 [M+H−H_2_O]^+^, 88.0391 [M+H−H_2_O−CO]^+^, 74.0236 [M+H−H_2_O−CH_2_CO]^+^
18	Asparagine (N)	C_4_H_8_N_2_O_3_	8.11	132.0535	133.0608	303.1093 (303.1088)	87.0552 [M+H−H_2_O−CO]^+^, 70.0287 [M+H−H_2_O−CO−NH_3_]^+^
19	Glutamic acid (E)	C_5_H_9_NO_4_	10.88	147.0532	148.0604	318.1089 (318.1084)	130.0497 [M+H−H_2_O]^+^, 102.0548 [M+H−H_2_O−CO]^+^, 84.0443 [M+H−2H_2_O−CO]^+^, 56.0497 [M+H−2H_2_O−2CO]^+^
20	Serine (S)	C_3_H_7_NO_3_	9.03	105.0426	106.0499	276.0983 (276.0979)	88.0393 [M+H−H_2_O]^+^, 70.0287 [M+H−2H_2_O]^+^
21	Azetidine-2-carboxylic acid (Aze)	C_4_H_7_NO_2_	10.97	101.0477	102.055	272.1035 (272.103)	84.0449 [M+H−H_2_O]^+^, 56.0500 [M+H−H_2_O−CO]^+^
22	Hypoglycin A	C_7_H_11_NO_2_	21.59	141.0790	142.0863	312.1348 (312.1343)	124.0762 [M+H−H_2_O]^+^, 96.0813 [M+H−H2O−CO]^+^, 74.0240, 67.0548
23	γ-Aminobutyric acid (GABA)	C_4_H_9_NO_2_	12.55	103.0633	104.0706	274.1192 (274.1186)	86.0601 [M+H−H_2_O]^+^, 87.0440 [M+H−NH_3_]^+^
24	L-3,4-Dihydroxyphenylalanine (L-DOPA)	C_9_H_11_NO_4_	15.68	197.0688	198.0761	368.1250 (368.1241)	181.0502 [M+H−NH_3_]^+^, 152.0709 [M+H−H_2_O−CO]^+^, 163.0399 [M+H−H_2_O−NH_3_]^+^, 135 [M+H−H_2_O−NH_3_−CO]^+^, 107.0492 [[M+H−H_2_O−NH_3_−2CO]^+^
25	L-Canavanine	C_5_H_12_N_4_O_3_	8.11	176.0909	177.0982	347.1465 (347.1462)	160.0726 [M+H−NH_3_]^+^, 118.0850, 102.0513
26	5-Hydroxy-pipecolic acid	C_6_H_11_NO_3_	11.86	145.0739	146.0812	316.1296 (316.1292)	128.0706 [M+H−H_2_O]^+^, 100.0759[M+H−H_2_O−CO]^+^, 82.0654 [M+H−2H_2_O−CO]^+^, 55.0547
27	Baikiain isomer	C_6_H_9_NO_2_	16.87	127.0633	128.0706	298.1194 (298.1186)	110.0595[M+H−H_2_O]^+^, 82.0658[M+H−H_2_O−CO]^+^, 80.0496, 74.0243, 65.0387, 55.0542
28	Baikiain	C_6_H_9_NO_2_	17.21	127.0633	128.0706	298.1192 (298.1186)	110.0595[M+H−H_2_O]^+^, 82.0655[M+H−H_2_O−CO]^+^, 80.0491, 74.0242, 65.0381, 55.054
29	Pipecolic acid	C_6_H_11_NO_2_	17.79	129.0790	130.0863	300.1345 (300.1343)	112.0756 [M+H−H_2_O]^+^, 84.0811(C_4_H_6_NO) [M+H−H_2_O−CO]^+^, 56.0502 (C_2_H_2_NO)
30	3-Hydroxymethyl tyrosine	C_10_H_13_NO_4_	16.17	211.0845	212.0917	382.1402 (382.1397)	194.0810 [M+H−H_2_O]^+^, 177.0525 [M+H−H_2_O−NH_3_]^+^, 148.0739, 91.0528, 77.0372, 55.0168
31	3-Hydroxymethyl phenylalanine	C_10_H_13_NO_3_	18.59	195.0895	196.0968	366.1452 (366.1448)	178.0847 [M+H−H_2_O]^+^, 103.0536 [M+H−NH_3_−H_2_O−CO]^+^, 91.0543, 77.0389
32	2,3 Diaminopropionic acid (DAPA)	C_3_H_8_N_2_O_2_	13.33	104.0586	105.0659	275.1151 (275.1139) 445.1622 * (445.1619)	88.0394 [M+H−NH_3_]^+^, 87.0553 [M−H−H_2_O]^+^
33	2,4-Diaminobutyric acid (DABA)	C_4_H_10_N_2_O_2_	13.66	118.0742	119.0815	289.1312 (289.1295) 459.1783 * (459.1775)	101.0714 [M+H−H_2_O]^+^, 102.0586 [M+H−NH_3_]^+^
34	Homoarginine	C_7_H_16_N_4_O_2_	10.1	188.1273	189.1346	359.1836 (359.1826)	172.1089 [M+H−NH_3_]^+^, 144.1138 [M+H−NH_3_−CO]^+^, 84.0801, 67.0542, 56.0499, 51.0243
35	4-Aminobenzoic acid (PABA)	C_7_H_7_NO_2_	20.4	137.0477	138.055	308.1035 (308.1030)	120.0445 [M+H−H_2_O]^+^, 94.0660, 92.0494 [M+H−H_2_O−CO]^+^, 75.0241 [M+H−H_2_O−CO−NH_3_]^+^, 77.0310, 65.0393
36	Theanine	C_7_H_14_N_2_O_3_	12.4	174.1004	175.1077	345.1555 (345.1557)	158.0826 [M+H−NH_3_]^+^, 130.0499 [M+H−NH_2_CH_2_CH_3_]^+^, 84.0445, 56.0485
37	L-Citrulline	C_6_H_13_N_3_O_3_	10.4	175.0957	176.103	346.1513 (346.1510)	159.0770 [M+H−NH_3_]^+^, 113.0706 [M+H−H_2_O−CO−NH_3_]^+^, 70.0656
38	L-Norvaline	C_5_H_11_NO_2_	18.8	117.0790	118.0863	288.1346 (288.1343)	110.0285 [M+H−H_2_O]^+^, 72.0799, 55.0538
39	Tranexamic acid	C_8_H_15_NO_2_	19.3	157.1103	158.1176	328.1657 (328.1656)	141.0916 [M+H−NH_3_]^+^, 140.1075 [M+H−H_2_O]^+^, 123.0810,112.1126, 95.0861, 77.0389, 67.0541, 55.0540
40	L-Norleucine	C_6_H_13_NO_2_	23.1	131.0946	132.1019	302.1499 (302.1499)	114.0916 [M+H−H_2_O]^+^, 86.0969, 77.0389, 69.0696
41	5-Hydroxy-L-tryptophan	C_11_H_12_N_2_O_3_	17.1	220.0848	221.0921	391.1403 (391.1401)	204.0668 [M+H−NH_3_]^+^, 186.0560, 162.0563, 116.0502, 107.0501, 77.0384
42	L-Homoserine	C_4_H_9_NO_3_	9.3	119.0582	120.0655	290.1138 (290.1135)	102.0534 [M+H−H_2_O]^+^, 74.0581, 56.0479
43	L-Ornithine	C_5_H_12_N_2_O_2_	14.8	132.0899	133.0972	473.1938 * (473.1932)	116.0705 [M+H−NH_3_]^+^, 115.0865 [M+H−H_2_O]^+^, 70.0651 [M+H−NH_3_−H_2_O−CO]^+^
44	*trans*-4-Hydroxy-L-proline	C_5_H_9_NO_3_	7.2	131.0582	132.0655	302.1136 (302.1135)	114.0563 [M+H−H_2_O]^+^, 86.0600, 68.0500, 58.0652
45	*N*-(2-Aminoethyl) glycine (AEG))	C_4_H_10_N_2_O_2_	13.5	118.0742	119.0815	459.1777 * (459.1775)	102.0533 [M+H−H_2_O]^+^, 56.0477
46	β-Methylamino-L-alanine (L-BMAA)	C_4_H_10_N_2_O_2_	13.3	118.0742	119.0815	459.1776 * (459.1775)	102.0554 [M+H−H_2_O]^+^, 58.0646
47	L-Canaline	C_4_H_10_N_2_O_3_	14.1	134.0691	135.0764	475.1727 * (475.1724)	118.0508 [M+H−NH_3_]^+^, 89.0709, 72.0444, 56.0495
48	β-ODAP	C_5_H_8_N_2_O_5_	8.5	176.0433	177.0506	347.0989 (347.0986)	160.0254 [M+H−NH_3_]^+^, 133.0607, 131.0448, 116.0341, 105.0655, 87.0551, 67.0289, 59.0602, 57.0444
49	4-Amino-L-phenylalanine	C_9_H_12_N_2_O_2_	10.7	180.0899	181.0972	351.1455 (351.1452)	164.0677 [M+H−NH_3_]^+^, 135.0886, 118.0612, 106.0620, 94.0620, 91.0504, 77.0350
50	β-Cyanoalanine	C_4_H_6_N_2_O_2_	10.5	114.0429	115.0502	285.0986 (285.0982)	89.0471 [M+H−CN]^+^, 74.0237, 69.0447 [M+H−H2O−CO]^+^ (CN=26.0031)
51	Ibotenic acid	C_5_H_6_N_2_O_4_	10.2	158.0328	159.04	329.0885 (329.0880)	113.0345 [M+H−H_2_O−CO]^+^, 94.0406, 67.0291, 68.0139, 56.0132, 52.0181
52	Muscinol	C_4_H_6_N_2_O_2_	12.05	114.0429	115.0502	285.0986 (285.0982)	98.0237 [M+H−NH_3_]^+^, 67.0216
53	L-2-Amino-adipic acid	C_6_H_11_NO_4_	13.25	161.0688	162.0761	332.1245 (332.1241)	144.0658 [M+H−H_2_O]^+^, 127.0411 [M+H−H_2_O−NH_3_]^+^, 116.0705 [M+H−H_2_O−CO]^+^, 55.0173
54	*O*-Acetyl-L-Serine	C_5_H_9_NO_4_	13.12	147.0532	148.0604	318.1087 (318.1084)	130.0498 [M+H−H_2_O]^+^, 131.0347 [M+H−NH_3_]^+^, 106.0457, 88.0392, 70.0295, 60.0450
55	*S-*Methyl-L-cysteine	C_4_H_9_NO_2_S	15.35	135.0354	136.0427	306.0908 (306.0907)	119.0124 [M+H−NH_3_]^+^, 77.0040, 73.0232
56	Cystine (L-cysteine derivative and a non-proteinogenic L-alpha-amino acid)	C_6_H_12_N_2_O_4_S_2_	16.1	240.0238	241.0311	581.1271 * (581.1271)	224.0046 [M+H−NH_3_]^+^, 205.9940 [M+H−NH_3_−H_2_O]^+^, 195.0256 [M+H−H_2_O−CO]^+^, 177.9991 [M+H−H_2_O−CO−NH_3_]^+^, 151.9835 [M+H−C_3_H_7_NO_2_]^+^, 122.027 [M+H−C_3_H_5_NO_2_S], 74.0236 [M+H−C_3_H_7_NO_2_−CH_2_S_2_]^+^
57	4-Carboxy-phenylalanine	C_10_H_11_NO_4_	18.83	209.0688	210.0761	380.1241 (380.1241)	164.0701, 146.0596, 103.0544, 91.0542, 77.0389
58	Taurine	C_2_H_7_NO_3_S	8.75	125.0147	126.0219	296.0702 (296.0699)	80.9645 [SO_3_]
59	4-Hydroxy-L-isoleucine	C_6_H_13_NO_3_	15.44	147.0895	148.0968	318.1456 (318.1448)	132.1015, 86.0964 [M+H−H_2_O−CO]^+^, 69.0699 [M+H−H_2_O−CO−NH_3_]^+^
60	γ-Glutamyl phenylalanine	C_14_H_18_N_2_O_5_	20.45	294.1216	295.1288	465.1770 (465.1768)	166.0852, 120.0797 [M+H−H_2_O−CO]^+^, 103.0535 [M+H−H_2_O−CO−NH_3_]^+^, 93.0694, 91.0537
61	3-Hydroxy norvaline	C_5_H_11_NO_3_	14.6	133.0739	134.0812	304.1299 (304.1292)	116.0700, 88,0758, 70.0651
62	2,6 Diaminopimelic acid (DAP)	C_7_H_14_N_2_O_4_	12.99	190.0954	191.1026	361.1510 (531.1986)	128.0731, 82.0671
63	4-Amino-3-hydroxy butyric acid	C_4_H_9_NO_3_	10.17	119.0582	120.0655	290.1141 (290.1135)	102.0541, 84.0440
64	*N*-Acetyl-L-ornithine	C_7_H_14_N_2_O_3_	12.19	174.1004	175.1077	345.1560 (345.1557)	158.0819, 157.0967, 133.0970, 115.0862, 116.0735, 70.0651
65	γ-Glutamyl-leucine	C_11_H_20_N_2_O_5_	19.32	260.1372	261.1445	431.1931 (431.1925)	244.1180, 198.1126, 132.1017, 130.0494, 86.0958, 84.0442, 56.0494
66	γ-Glutamyl-tyrosine	C_14_H_18_N_2_O_6_	15.28	310.1165	311.1238	481.1718 (481.1718)	294.0975, 248.0915, 202.0855, 182.0804, 165.0539, 147.0429, 136.0750, 130.0495, 123.0426, 119.0488, 95.0487, 91.0529, 84.0435
67	L-Mimosine	C_8_H_10_N_2_O_4_	8.98	198.0641	199.0713	369.1198 (369.1193)	112.0395 (Dihydroxypyridine), 94.0304, 79.0218
68	N^5^-Methylglutamine	C_6_H_12_N_2_O_3_	10.63	160.0848	161.0921	331.1405 (331.1401)	144.0652 [M+H−NH_3_], 130.0502 [M+H−CH_3_NH_2_], 115.0869 [M+H−H_2_O−CO], 84.0448 [M+H−CH_3_NH_2_−H_2_O−CO], 56.0497 [M+H−CH_3_NH_2_−H_2_O−2CO]
69	(S)-Willardiine	C_7_H_9_N_3_O_4_	9.56	199.0593	200.0666	370.1148 (370.1146)	191.1031, 175.1081, 161.0920, 154.0617, 144.0654, 130.0500, 115.0869, 84.0448
70	4-Hydroxy-pipecolic acid	C_6_H_11_NO_3_	9.51	145.0739	146.0812	316.1297 (316.1292)	128.0701 [M+H−H_2_O]^+^, 100.0755[M+H−H_2_O−CO]^+^, 82.0654 [M+H−2H_2_O−CO]^+^, 56.0508, 55.0551
71	DL-5-Hydroxylysine	C_6_H_14_N_2_O_3_	12.2	162.1004	163.1077	503.2038 * (503.2037)	145.0969 [M+H−H_2_O]^+^, 128.0702 [M+H−H_2_O−NH_3_]^+^, 100.0758 [M+H−H_2_O−NH_3_−CO]^+^, 82.0652, 67.0439, 56.0494, 55.0542
72	α-Aminobutyric acid (AABA)	C_4_H_9_NO_2_	14.7	103.0633	104.0706	274.1187 (274.1186)	86.0590 [M+H−H_2_O]^+^
73	β-Aminobutyric acid (BABA)	C_4_H_9_NO_2_	12.53	103.0633	104.0706	274.1185 (274.1186)	58.0645 [M+H−H_2_O−CO]^+^
74	β-Aminoisobutyric acid (BIABA)	C_4_H_9_NO_2_	13.56	103.0633	104.0706	274.1180 (274.1186)	58.0639 [M+H−H_2_O−CO]^+^
75	Tryptamine	C_10_H_12_N_2_	27.1	160.1000	161.1073	331.1558 (331.1553)	144.0814 [M+H−NH_3_]^+^ (C_10_H_10_N), 127.0546 [M+H−2NH_3_]^+^ (C_10_H_8_), 117.0689 (C_7_H_5_N_2_), 115.0580 (C_7_H_3_N_2_), 91.0580 (C_5_H_3_N_2_)
76	PEA (phenethylamine)	C_8_H_11_N	26.2	121.0891	122.0964	292.1452 (292.1444)	105.0706 [M+H−NH_3_]^+^, 77.0377, 51.0224

Note: * = double derivatization; ** theoretical accurate mass; and in all samples analyzed, two common fragment ions detected were 6-aminoquinoline carbonyl group, *m*/*z* 171.0558 [M + H]^+^ C_10_H_7_N_2_O and 6-aminoquinoline (AMQ), *m*/*z* 145.076 [M + H]^+^ C_9_H_9_N_2_.

**Table 4:** Screening of free proteinogenic and non-proteinogenic amino acids in 22 distinct legume water-soaked samples.

#	Compound Name	RT	AQC Tag Mass	1	2	3	4	5	6	7	8	9	10	11	12	13 *	14	15	16	17	18	19 *	20	21 *	22 *	Detected Samples/ Total Number
Essential Free Protein Amino Acids																										
1	Histidine	7.1	326.1248	L	L	L	L	L	M	L	L	L	L	L	L	M	L	M	L	L	L	M	L	L	-	21/22
2	Isoleucine	21.87	302.1499	M	M	M	M	M	H	H	M	M	M	M	M	M	M	M	M	M	M	M	M	H	H	22/22
3	Leucine	22.34	302.1499	H	M	H	H	H	H	H	H	M	M	H	H	M	H	H	H	H	H	M	H	H	H	22/22
4	Lysine	16.43	487.2088	H	L	M	H	H	H	H	H	M	M	H	H	L	H	H	H	H	H	M	M	H	-	21/22
5	Methionine	17.91	320.1063	L	L	L	L	L	L	L	L	L	L	L	L	L	L	L	L	L	L	L	L	L	L	22/22
6	Phenylalanine	23.4	336.1343	M	M	M	H	M	H	H	H	M	M	H	M	M	L	M	M	M	H	M	M	M	M	22/22
7	Threonine	11.24	290.1135	M	M	M	M	M	M	M	M	M	M	M	M	M	M	M	M	M	M	M	M	M	M	22/22
8	Tryptophan	24.37	375.1452	H	L	M	L	M	L	L	L	L	L	L	L	M	M	L	M	L	L	L	M	L	L	22/22
9	Valine	18.11	288.1343	H	H	H	M	M	H	H	M	L	M	H	M	H	M	M	M	H	M	M	M	M	M	22/22
Conditionally Essential Free Protein Amino Acids																										
10	Arginine	8.4	345.167	M	M	M	H	H	H	H	H	H	H	M	H	H	H	H	H	M	M	M	H	H	L	22/22
11	Cysteine	13.90	292.075	L	L	L	L	L	L	L	L	L	L	L	L	L	L	L	L	-	L	L	L	-	-	19/22
12	Glutamine	9.01	317.1244	M	M	M	M	M	M	M	M	M	M	M	M	M	M	M	M	M	M	H	M	M	-	21/22
13	Glycine	9.59	246.0873	M	H	M	M	M	M	M	M	M	M	M	M	M	M	M	M	M	M	M	M	M	H	22/22
14	Proline	13.34	286.1186	M	H	M	M	M	M	M	M	H	H	M	H	H	H	M	M	H	M	H	H	M	M	22/22
15	Tyrosine	17.61	352.1292	L	M	M	M	L	M	M	M	L	L	L	M	M	M	M	M	M	M	M	M	H	M	22/22
Non-Essential Free Protein Amino Acids																										
16	Alanine	12.21	260.103	H	M	H	M	M	M	M	M	H	H	M	H	H	M	M	M	M	M	H	M	M	M	22/22
17	Aspartic acid	10.27	304.0928	H	H	H	H	H	H	H	H	H	H	H	H	H	H	H	H	H	H	H	H	H	H	22/22
18	Asparagine	8.10	303.1088	M	M	M	M	M	M	M	M	M	M	M	M	H	M	M	M	M	M	L	M	M	-	21/22
19	Glutamic acid	10.87	318.1084	H	H	H	H	H	H	H	H	H	H	H	H	H	H	H	H	H	H	H	H	H	H	22/22
20	Serine	9.01	276.0979	M	M	M	M	M	M	M	M	M	M	M	M	M	M	M	M	H	M	M	M	M	M	22/22
Non-Proteinogenic Amino Acids (NPAAs)																										
21	Pipecolic acid	17.75	300.1343	H	H	H	H	-	L	M	H	T	L	H	H	M	-	L	L	L	L	T	T	-	-	18/22
22	γ-Glutamyl-leucine	19.33	431.1925	M	H	M	L	L	M	M	H	H	L	L	M	-	L	T	-	L	-	L	-	-	-	16/22
23	γ-Acetyl-L-ornithine	12.19	345.1557	L	L	L	L	L	M	L	L	M	T	L	M	M	M	-	-	L	-	-	-	-	-	15/22
24	γ-Aminobutyric acid (GABA)	12.55	274.1192	H	H	H	H	H	H	H	H	H	H	H	H	L	H	H	H	M	H	L	H	M	T	22/22
25	5-Hydroxynorvaline	11.4	304.1292	M	M	L	L	-	-	-	-	-	-	-	L	-	-	-	-	-	M	-	-	-	-	6/22
26	γ-Glutamyl-phenyl-L-alanine	20.45	465.1768	L	L	L	L	L	L	L	M	M	L	T	H	-	L	H	-	-	H	L	M	L	L	19/22
27	Homoserine	9.3	290.1135	M	L	L	H	H	-	-	-	-	-	-	-	T	H	H	H	M	M	-	-	-	-	11/22
28	α-Aminoadipic acid	13.2	332.1241	L	L	L	L	M	M	L	L	L	L	L	M	L	L	M	M	L	L	L	L	L	H	22/22
29	*N*-Methylglutamine	10.63	331.1401	-	-	-	-	M	-	-	-	-	-	-	-	-	M	-	-	-	M	-	-	-	-	3/22
30	α-Amino-β-acetylaminopropionic acid	9.8	317.1244	-	-	-	-	M	-	-	-	-	-	-	-	-	-	-	-	-	-	-	-	-	-	1/22
31	β-Aminoisobutyric acid (BAIBA)	13.58	274.1186	-	-	-	-	-	-	-	-	-	-	-	M	-	-	-	-	-	-	-	-	-	-	1/22
32	α-Aminobutyric acid (Homoalanine) (AABA)	14.87	274.1186	T	T	T	T	-	-	-	-	-	-	-	T	-	-	-	-	-	-	-	-	-	-	5/22
33	γ-Glutamyl-tyrosine	15.28	481.1718	-	-	-	-	-	-	-	-	-	-	-	M	-	-	H	T	-	H	-	M	-	-	5/22
34	Baikiain	17.18	298.1186	-	-	-	-	-	-	-	-	-	-	-	-	H	-	-	-	-	-	-	-	-	-	1/22
35	Baikiain isomer	16.89	298.1186	-	-	-	-	-	-	-	-	-	-	-	-	M	-	-	-	-	-	-	-	-	-	1/22
36	Hydroxy-L-proline	7.2	302.1135	-	-	-	-	-	-	-	-	-	-	-	-	T	-	-	-	-	-	-	T	-	-	2/22
37	4-Hydroxypipecolic acid	9.49	316.1292	-	-	-	-	-	-	-	-	-	-	-	-	M	-	-	-	-	-	-	-	-	-	1/22
38	5-Hydroxypipecolic acid	11.86	316.1292	-	-	-	-	-	-	-	-	-	-	-	-	L	-	-	-	-	-	-	-	-	-	1/22
39	3-Hydroxymethyl phenylalanine	18.57	366.1448	-	-	-	-	-	-	-	-	-	-	-	-	M	-	-	-	-	-	-	-	-	-	1/22
40	3-Hydroxymethyl tyrosine	16.2	382.1402	-	-	-	-	-	-	-	-	-	-	-	-	H	-	-	-	-	-	-	-	-	-	1/22
41	*m*-Carboxy-phenylalanine	19.84	380.1241	-	-	-	-	-	-	-	-	-	-	-	-	M	-	-	-	-	-	-	-	-	-	1/22
42	γ-Hydroxyornithine	6.86	319.1401	-	-	-	-	-	-	-	-	-	-	-	-	-	M	-	-	-	-	-	-	-	-	1/22
43	Homoarginine	10.1	359.1826	-	-	-	-	-	-	-	-	-	-	-	-	-	L	-	-	H	L	-	-	-	-	3/22
44	γ-Hydroxyarginine	7.03/7.27	361.1619	-	-	-	-	-	-	-	-	-	-	-	-	-	M	-	T	-	-	-	-	-	-	2/22
45	γ-Hydroxy-L-homoarginine	7.8	375.1775	-	-	-	-	-	-	-	-	-	-	-	-	-	M	-	-	M	-	-	-	-	-	2/22
46	l-3,4-Dihydroxy-phenylalanine (L-DOPA)	15.68	368.1241	-	-	-	-	-	-	-	-	-	-	-	-	-	-	-	H	-	-	L	-	H	-	3/22
47	Deoxyfructose L-dopa (L-DOPA hexose isomer)	14.52	530.1769	-	-	-	-	-	-	-	-	-	-	-	-	-	-	-	H	-	-	-	-	-	-	1/22
48	β-*N*-Oxalyl-L-R, β-diaminopropionic acid (β-ODAP)	8.5	347.0986	-	-	-	-	-	-	-	-	-	-	-	-	-	-	-	-	H	-	-	-	-	-	1/22
49	α-*N*-Oxalyl-L-R, β-diaminopropionic acid (α-ODAP, less toxic than β-ODAP)	9.22	347.0986	-	-	-	-	-	-	-	-	-	-	-	-	-	-	-	-	H	-	-	-	-	-	1/22
50	γ-*N*-Oxalyl-L-α, γ-diaminobutyric acid (γ-ODAB)	8.8	361.1142	-	-	-	-	-	-	-	-	-	-	-	-	-	-	-	-	L	-	-	-	-	-	1/22
51	2,3 Diaminopropionic acid (DAPA)	13.3	445.1619	-	-	-	-	-	-	-	-	-	-	-	-	-	-	-	-	-	L	-	-	-	-	1/22
52	2,4-Diaminobutyric acid (DABA)	13.66	459.1775	-	-	-	-	-	-	-	-	-	-	-	-	-	-	-	-	-	L	-	-	-	-	1/22
53	L-Canavanine	8.11	347.1462	-	-	-	-	-	-	-	-	-	-	-	-	-	-	-	-	-	H	-	-	-	-	1/22
54	Mimosine	8.98	369.1193	-	-	-	-	-	-	-	-	-	-	-	-	-	-	-	-	-	-	H	-	-	-	1/22
55	3-Carboxysalsolinol/N-acetyl-L-tyrosine	15.1/19.4	394.1397	-	-	-	-	-	-	-	-	-	-	-	-	-	-	-	-	-	-	-	-	M	-	1/22
56	L-3-Carboxy-6,7-dihydroxy-1,2,3,4-tetrahydroisoquinoline	16.78	380.1241	-	-	-	-	-	-	-	-	-	-	-	-	-	-	-	-	-	-	-	-	M	-	1/22
57	*N*-Methyl-L-DOPA	18.51	382.1397	-	-	-	-	-	-	-	-	-	-	-	-	-	-	-	M	-	-	-	-	M	-	2/22
58	γ-Ethylidene glutamic acid	16.08	344.1241	-	-	-	-	-	-	-	-	-	-	-	-	-	-	-	-	-	-	-	-	-	M	1/22
59	γ-Methylene glutamic acid (L-MDAM)	13.65	330.1084	-	-	-	-	-	-	-	-	-	-	-	-	-	-	-	-	-	-	-	-	-	M	1/22
60	Hydroxy-methyl glutamic acid	9.48	348.119	-	-	-	-	-	-	-	-	-	-	-	-	-	-	-	-	-	-	-	-	-	L	1/22
61	γ-Ethyl glutamic acid	15.74	346.1397	-	-	-	-	-	-	-	-	-	-	-	-	-	-	-	-	-	-	-	-	-	M	1/22
62	Taurine	8.7	296.0677	-	-	-	-	-	-	-	-	-	-	-	-	-	-	-	L	-	-	L	-	-	-	2/22
63	*S*-Methyl-L-cysteine	15.38	306.0907	H	H	H	H	L	H	H	H	H	H	T	L	-	L	L	L	L	-	-	L	-	-	17/22
64	γ-Glutamyl-*S*-methylcysteine (GMC)	13.75	435.1333	M	M	H	H	-	H	L	L	H	H	L	T	-	L	-	-	L	-	-	L	-	-	14/22
65	S-Methylhomoglutathione	13.89	506.1704	L	M	L	M	-	M	L	M	-	L	T	-	-	L	-	-	-	-	-	-	-	-	10/22
66	L-Homoglutathione	12.72	492.1547	L	T	L	-	-	L	L	M	-	M	L	M	-	-	-	-	-	-	-	-	-	-	9/22
67	γ-Glutamylmethionine (GGM)	15.75	449.1489	L	L	M	L	-	-	-	-	-	-	-	T	-	-	L	-	-	-	-	-	-	-	6/22

Note: (1) 1. Phaseolus vulgaris; 2. P. coccineus; 3. P. acutifolius; 4. P. lunatus; 5. Pisum sativum; 6. Vigna radiata; 7. V. mungo; 8. V. umbellata; 9. V. unguiculata; 10. V. aconitifolia; 11. V. angularis; 12. Cajanus Cajan; 13. Caesalpinia spinosa; 14. Lens culinaris; 15. Cicer arietinum; 16. Vicia faba; 17. Lathyrus sativus; 18. Medicago sativa; 19. Mimosa pudica; 20. Glycine max; 21. Mucuna pruriens; 22. Caesalpinia bonducella; (2) H = high (ToF-MS signal intensity >7 × 10^6^ cps); M = medium (signal intensity between 7 × 10^5^ to 7 × 10^6^ cps); L = low (signal intensity between 5 × 10^4^ to 7 × 10^5^ cps); and T = trace (signal intensity < 5 × 10^4^ cps). H, M, L, and T values were based on the analysis of compounds within the sample but not between the samples. (3) * Data shown for the powdered sample; [C. spinosa and C bonducella did not absorb water upon soaking]. (4) ’-‘ = not detected.

## Data Availability

Data available upon request.

## Supplementary Materials

The following supporting information can be downloaded at https://www.mdpi.com/article/10.3390/foods14040611/s1: Figure S1. Classification of legume samples based on family, sub-family, genus and species used for LC-MS analysis; Figure S2. Extracted ion chromatograms and mass spectrum for protein, and non-protein amino acids.
