# Supplementary material for: Comprehensive Profiling of Free Proteinogenic and Non-Proteinogenic Amino Acids in Common Legumes Using LC-QToF: Targeted and Non-Targeted Approaches
Source: Foods. 2025 Feb 12;14(4):611. doi: 10.3390/foods14040611 (PMC11854323; doi:10.3390/foods14040611)
Supplement: Supplementary file 1 [file foods-14-00611-s001.zip › foods-3441491-supplementary.pdf]

foods

Supporting Information

Comprehensive Profiling of Free Proteinogenic and Non-Proteinogenic Amino Acids in Common Legumes Using LC-QToF: Targeted and Non-Targeted Approaches

Bharathi Avula [1,*], Kumar Katragunta [1], Iffat Parveen [1], Kiran Kumar Tatapudi [1], Amar G. Chittiboyina [1], Yan-Hong Wang [1], and Ikhlas A. Khan [1,2,*]

[1] National Center for Natural Products Research, School of Pharmacy, University of Mississippi, University, MS 38677, USA; bavula@olemiss.edu (B.A.); kkatragu@olemiss.edu (K.K.); iparveen@olemiss.edu (I.P.); kktatapu@olemiss.edu (K.K.T.); amar@olemiss.edu (A.G.C.); wangyh@olemiss.edu (Y-H.W.)
[2] Division of Pharmacognosy, Department of BioMolecular Sciences, School of Pharmacy, University of Mississippi, University, MS 38677, USA; ikhan@olemiss.edu (I.A.K.)
* Correspondence: Correspondence: bavula@olemiss.edu; ikhan@olemiss.edu; Tel.: +1-662-915-7821

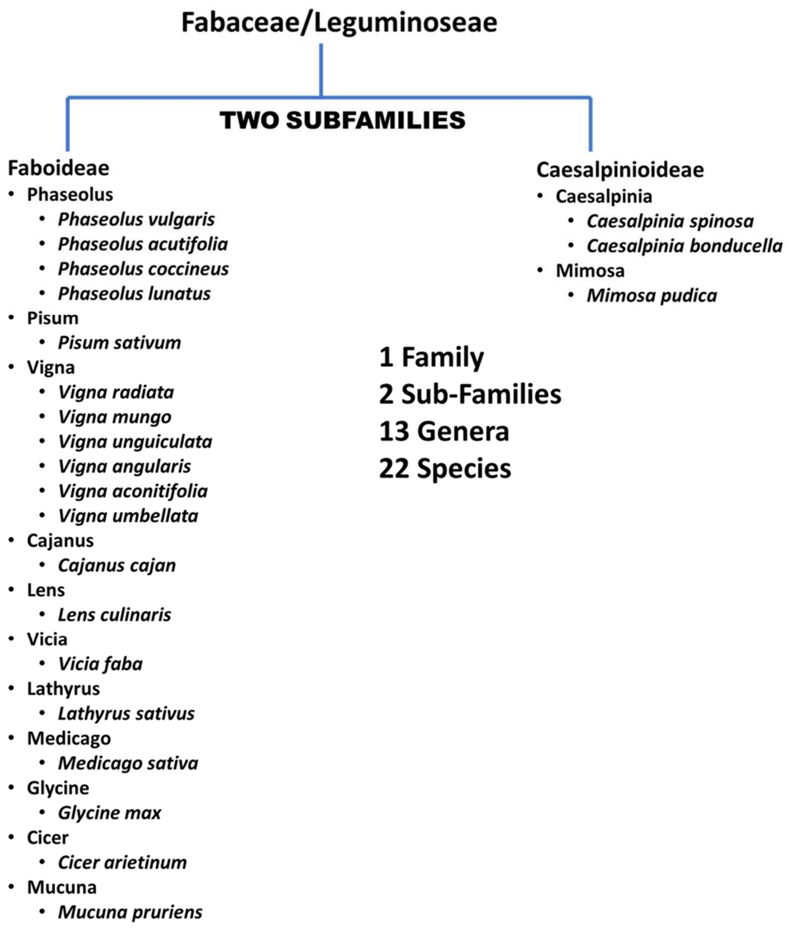

Figure 1. S. Classification of legume samples based on family, sub-family, genus and species used for LC-MS analysis.

Protein Amino Acids

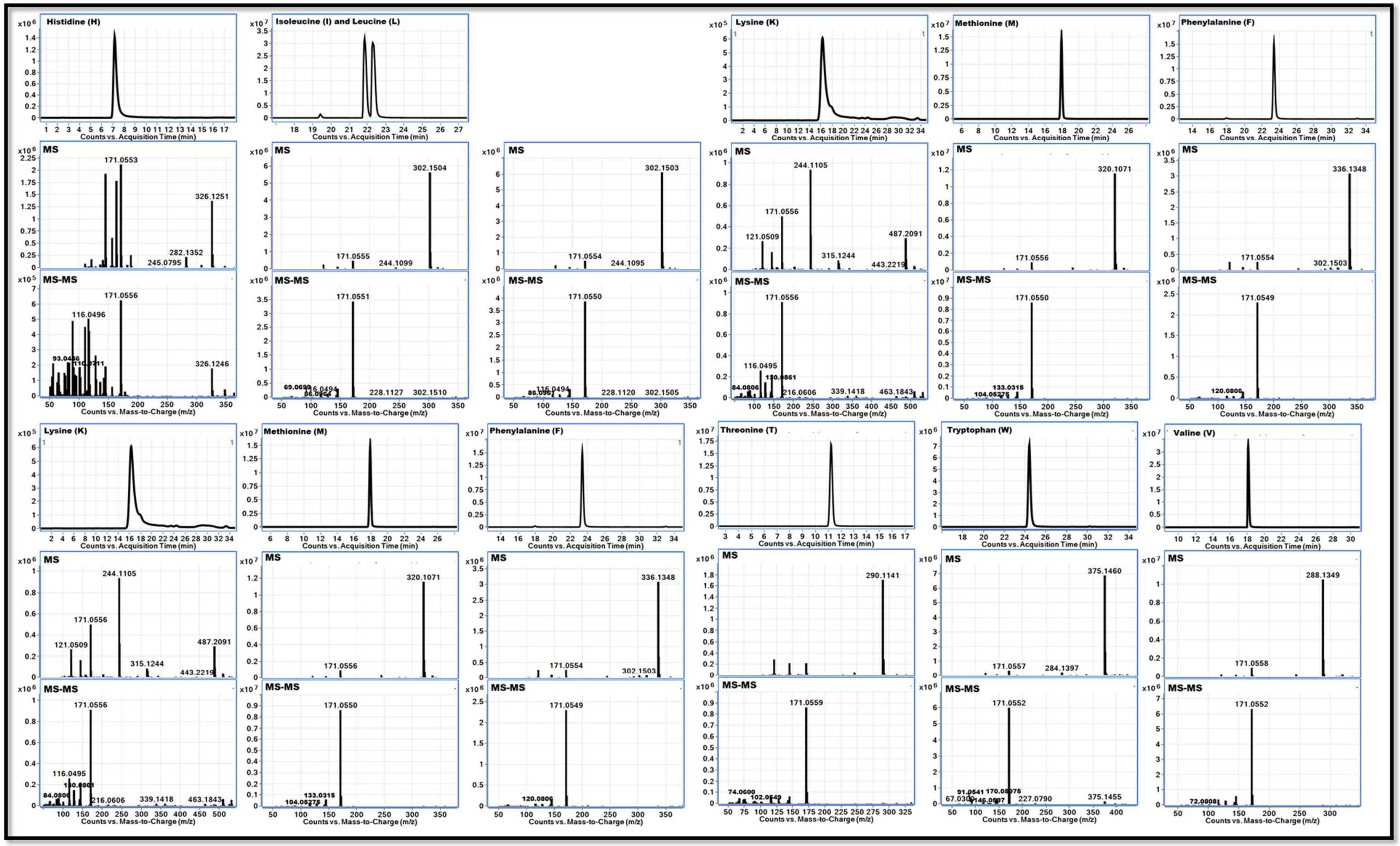

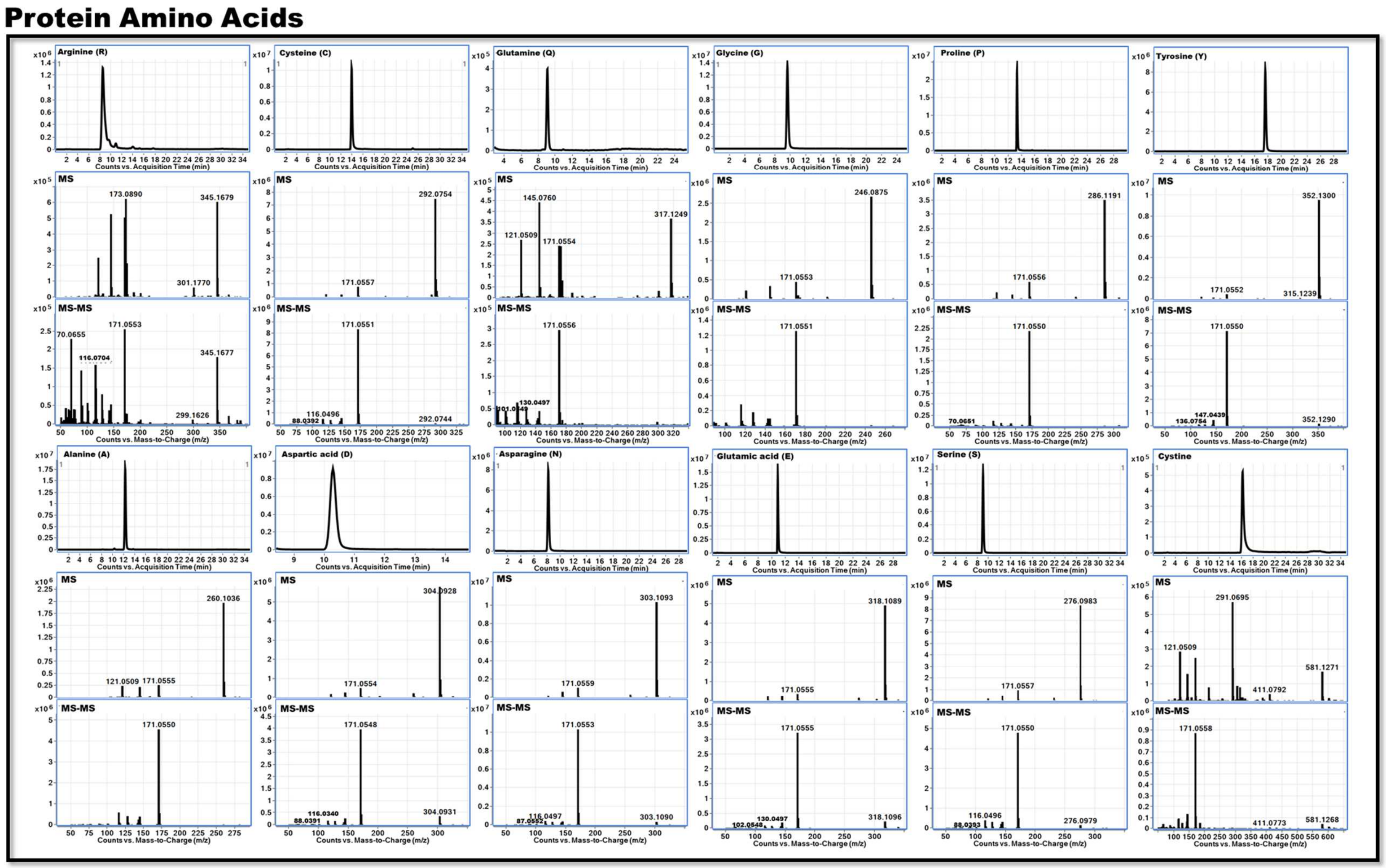
Protein Amino Acids
Arginine (R)
Cysteine (C)
Glutamine (Q)
Glycine (G)
Proline (P)
Tyrosine (Y)
Counts vs. Acquisition Time (min)
MS
173.0890
345.1679
301.1770
292.0754
171.0557
145.0760
317.1249
121.0509
171.0554
246.0875
171.0553
286.1191
171.0556
352.1300
171.0552
315.1239
MS-MS
70.0655
171.0553
116.0704
345.1677
299.1626
171.0551
116.0496
88.0392
292.0744
171.0556
130.0497
171.0551
171.0550
70.0651
147.0439
136.0754
352.1290
Counts vs. Mass-to-Charge (m/z)
Alanine (A)
Aspartic acid (D)
Asparagine (N)
Glutamic acid (E)
Serine (S)
Cystine
260.1036
121.0509
171.0555
304.0928
171.0554
303.1093
171.0559
318.1089
276.0983
171.0557
291.0695
581.1271
411.0792
171.0548
116.0340
88.0391
304.0931
116.0497
87.0552
303.1090
130.0497
102.0548
318.1096
116.0496
88.0393
276.0979
171.0558
411.0773
581.1268

Non-Protein Amino Acids

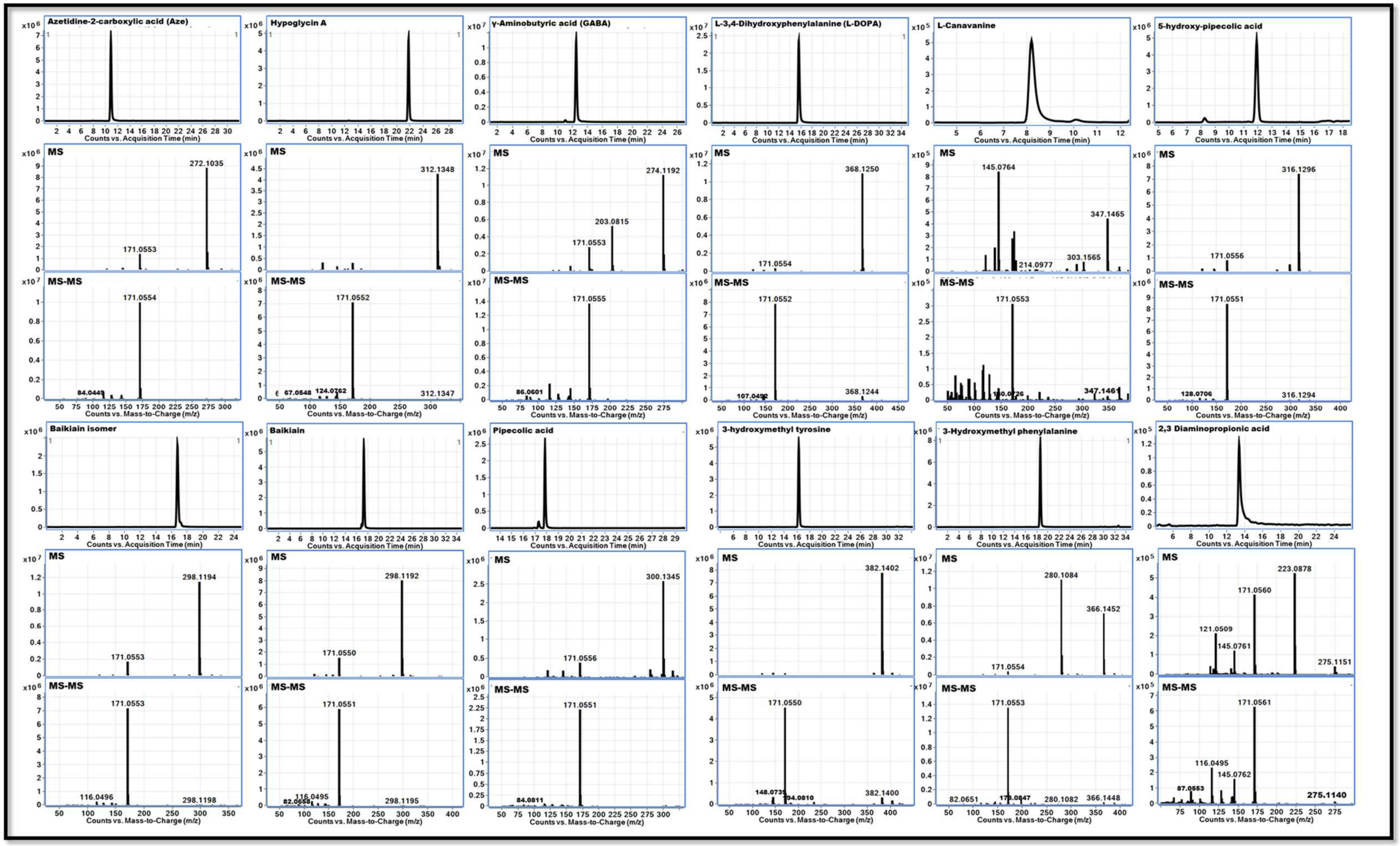

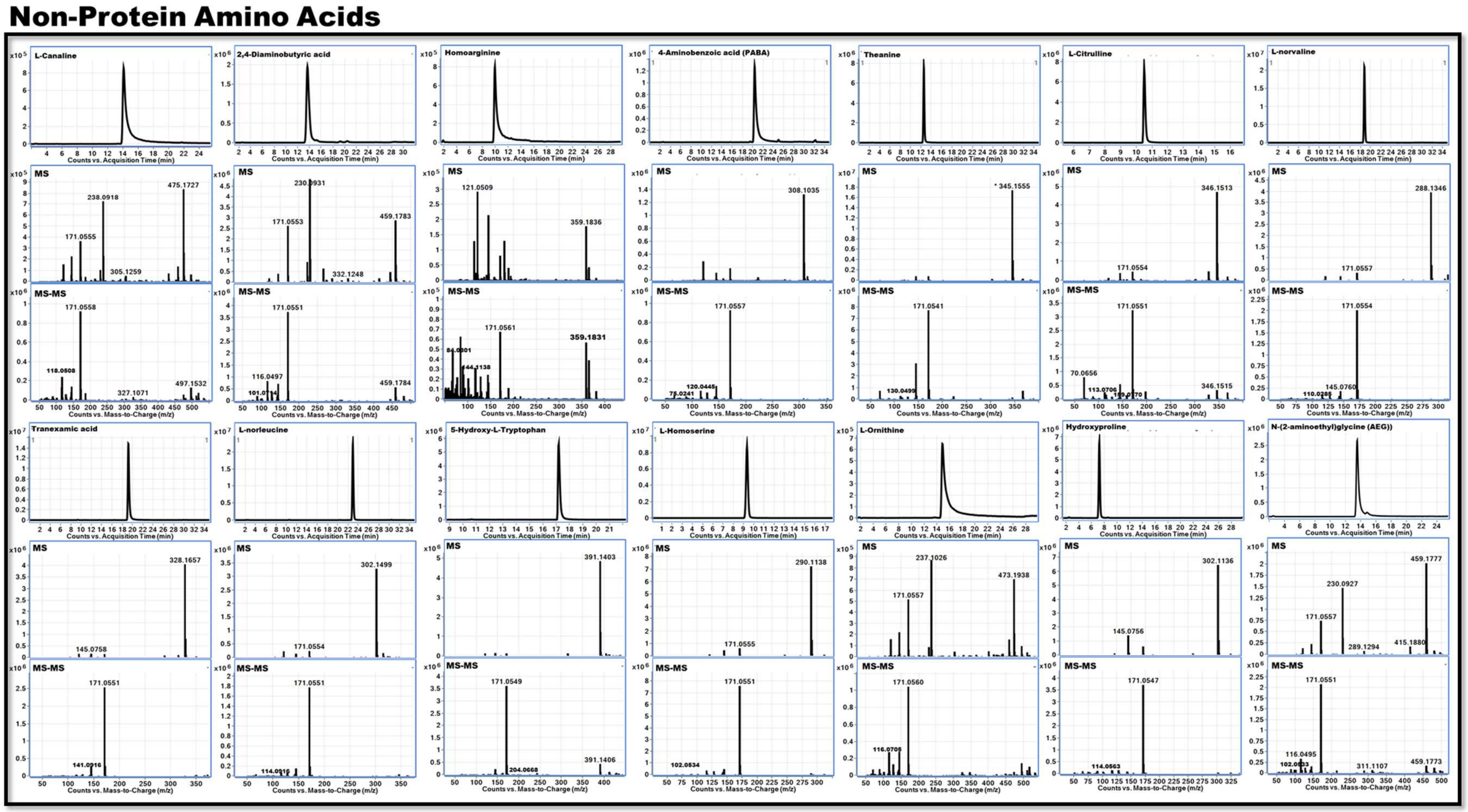
Non-Protein Amino Acids
L-Canaline
2,4-Diaminobutyric acid
Homoarginine
4-Aminobenzoic acid (PABA)
Theanine
L-Citrulline
L-norvaline
Counts vs. Acquisition Time (min)
MS
238.0918
171.0555
305.1259
475.1727
230.0931
171.0553
332.1248
459.1783
121.0509
359.1836
308.1035
345.1555
346.1513
171.0554
288.1346
171.0557
MS-MS
171.0558
118.0508
327.1071
497.1532
171.0551
116.0497
459.1784
171.0561
359.1831
120.0445
171.0557
171.0541
130.0499
70.0656
113.0706
346.1515
171.0554
145.0760
Counts vs. Mass-to-Charge (m/z)
Tranexamic acid
L-norleucine
5-Hydroxy-L-Tryptophan
L-Homoserine
L-Ornithine
Hydroxyproline
N-(2-aminoethyl)glycine (AEG)
328.1657
145.0758
302.1499
171.0554
391.1403
290.1138
171.0555
237.1026
171.0557
473.1938
302.1136
145.0756
459.1777
230.0927
289.1294
415.1880
171.0551
141.0916
171.0549
204.0668
391.1406
102.0534
171.0560
116.0705
171.0547
114.0563
116.0495
311.1107
459.1773

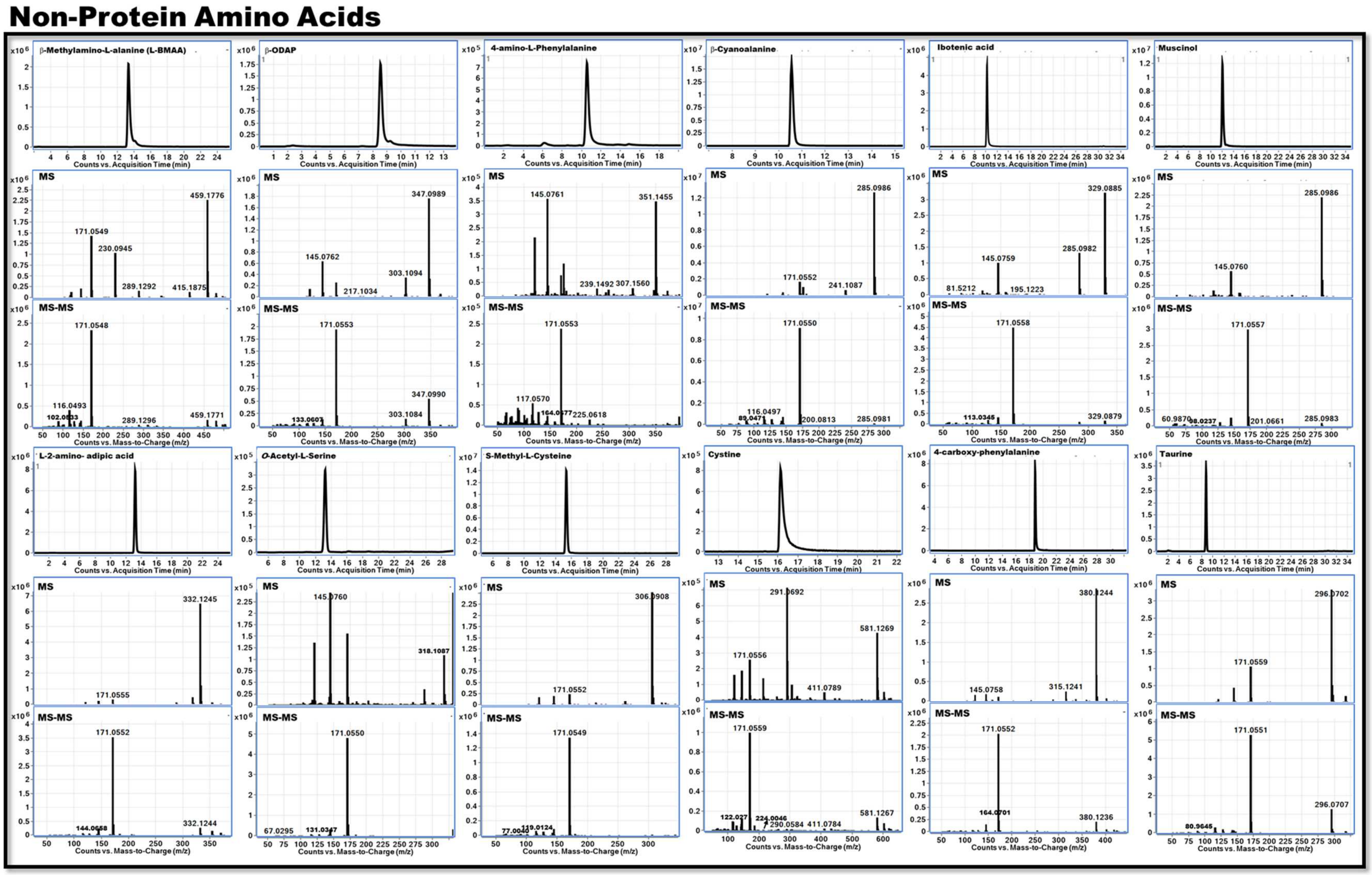
Non-Protein Amino Acids
β-Methylamino-L-alanine (L-BMAA)
β-ODAP
4-amino-L-Phenylalanine
β-Cyanoalanine
Ibotenic acid
Muscinol
L-2-amino- adipic acid
O-Acetyl-L-Serine
S-Methyl-L-Cysteine
Cystine
4-carboxy-phenylalanine
Taurine
MS
MS-MS
Counts vs. Acquisition Time (min)
Counts vs. Mass-to-Charge (m/z)
171.0549
230.0945
289.1292
415.1875
459.1776
145.0762
217.1034
303.1094
347.0989
145.0761
239.1492
307.1560
351.1455
171.0552
241.1087
285.0986
81.5212
145.0759
195.1223
285.0982
329.0885
145.0760
171.0548
116.0493
289.1296
459.1771
171.0553
303.1084
347.0990
117.0570
225.0618
171.0550
116.0497
200.0813
285.0981
171.0558
329.0879
171.0557
201.0661
285.0983
332.1245
171.0555
318.1087
171.0552
581.1269
411.0789
171.0556
315.1241
145.0758
171.0559
144.0558
332.1244
67.0295
131.0347
171.0549
77.0040
581.1267
411.0784
380.1236
164.0701
171.0551
80.9645
296.0707

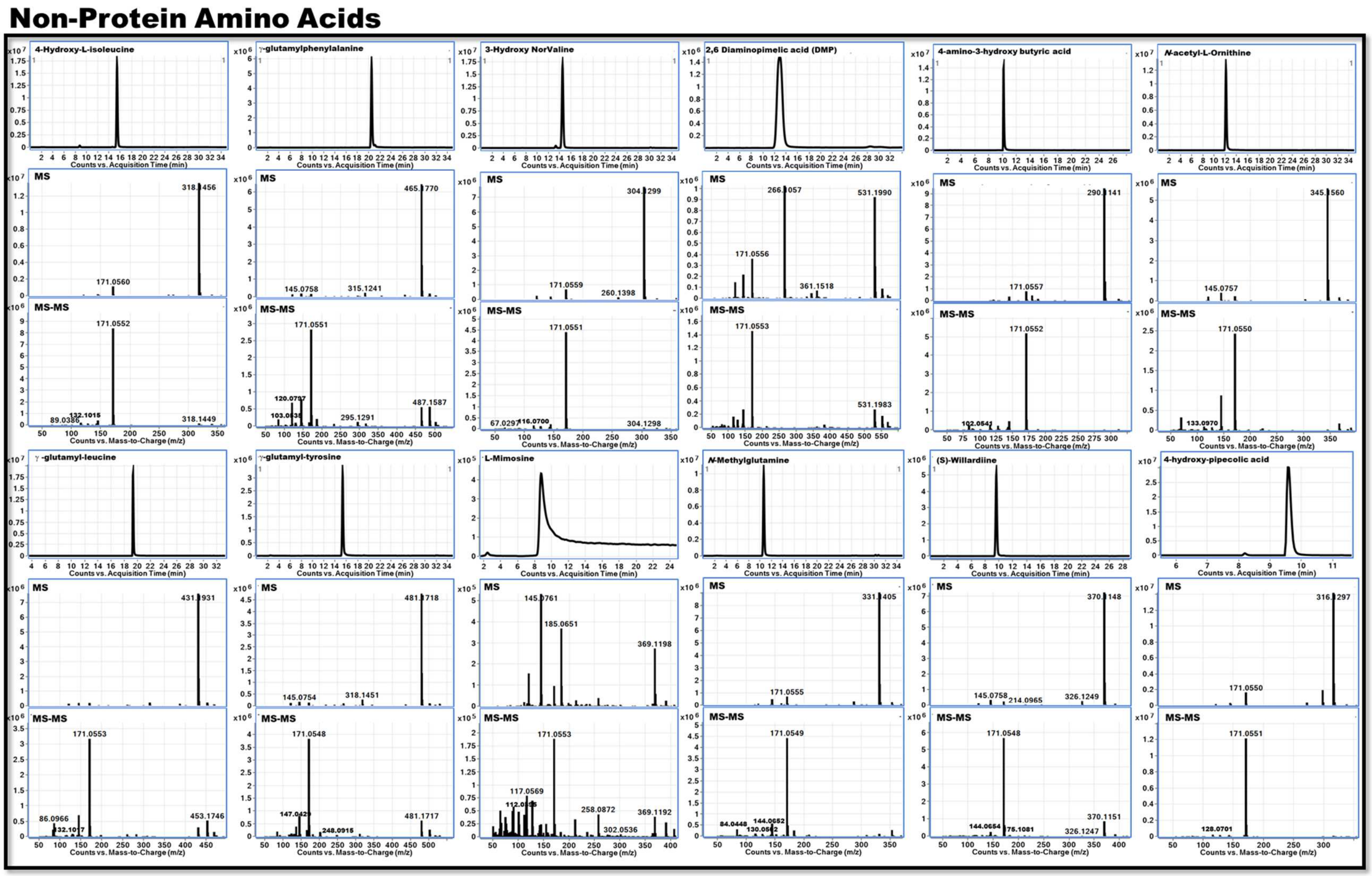
Non-Protein Amino Acids
4-Hydroxy-L-isoleucine
γ-glutamylphenylalanine
3-Hydroxy NorValine
2,6 Diaminopimelic acid (DMP)
4-amino-3-hydroxy butyric acid
N-acetyl-L-Ornithine
γ-glutamyl-leucine
γ-glutamyl-tyrosine
L-Mimosine
N-Methylglutamine
(S)-Willardiine
4-hydroxy-pipecolic acid
MS
MS-MS
Counts vs. Acquisition Time (min)
Counts vs. Mass-to-Charge (m/z)

Non-Protein Amino Acids

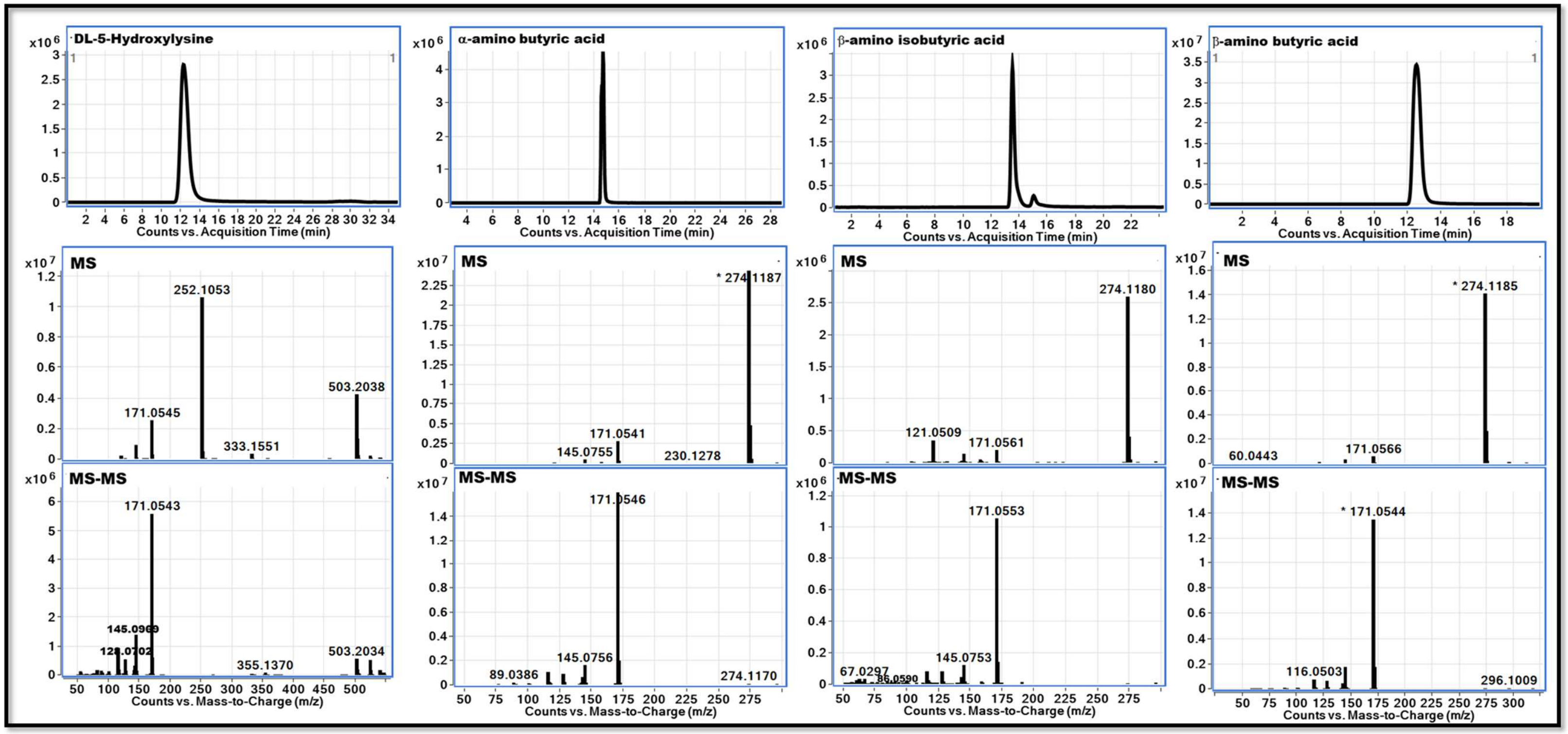

Figure S2. Extracted ion chromatograms and mass spectrum for protein, and non-protein amino acids.
